# Particle swarm optimization-based automatic parameter selection for deep neural networks and its applications in large-scale and high-dimensional data

**DOI:** 10.1371/journal.pone.0188746

**Published:** 2017-12-13

**Authors:** Fei Ye

**Affiliations:** School of information science and technology, Southwest Jiaotong University, ChengDu, China; Beihang University, CHINA

## Abstract

In this paper, we propose a new automatic hyperparameter selection approach for determining the optimal network configuration (network structure and hyperparameters) for deep neural networks using particle swarm optimization (PSO) in combination with a steepest gradient descent algorithm. In the proposed approach, network configurations were coded as a set of real-number m-dimensional vectors as the individuals of the PSO algorithm in the search procedure. During the search procedure, the PSO algorithm is employed to search for optimal network configurations via the particles moving in a finite search space, and the steepest gradient descent algorithm is used to train the DNN classifier with a few training epochs (to find a local optimal solution) during the population evaluation of PSO. After the optimization scheme, the steepest gradient descent algorithm is performed with more epochs and the final solutions (*pbest* and *gbest*) of the PSO algorithm to train a final ensemble model and individual DNN classifiers, respectively. The local search ability of the steepest gradient descent algorithm and the global search capabilities of the PSO algorithm are exploited to determine an optimal solution that is close to the global optimum. We constructed several experiments on hand-written characters and biological activity prediction datasets to show that the DNN classifiers trained by the network configurations expressed by the final solutions of the PSO algorithm, employed to construct an ensemble model and individual classifier, outperform the random approach in terms of the generalization performance. Therefore, the proposed approach can be regarded an alternative tool for automatic network structure and parameter selection for deep neural networks.

## 1. Introduction

The current growth in Internet information and computational hardware development, such as Facebook and other well-known business websites, is enabling a wide range of researchers to utilize various advanced techniques, such as machine learning tools, to capture available and important information and analyze this processed information to provide better business decisions for the government and industry. A critical challenge in the development of advanced techniques and application of them to process more complicated problems is how to select more appropriate models that fit the specific data collected from real-world applications well. Although machine learning approaches has been successfully applied in a wide range of pattern recognition and prediction tasks, limitations of these techniques in processing large-scale and high-dimensional data still exist. Typically, in most pattern recognition tasks, the data is usually collected from a group of digital images or action videos, and each image of these data contains a high-dimensional pixel vector. Convolution machine learning approaches, such as support vector machine (SVM), have difficulty processing such data due to their shallow structures, which fail to capture the high-level abstract feature representation from the high-dimensional row data. The above difficulties and challenges have inspired most research groups and companies to find and develop more advanced techniques to address such issues. Deep learning techniques are among the most popular and representative methods of these advanced techniques. In recent years, deep learning techniques have been widely developed and applied in many real-world applications due to their excellent performance on large-scale and high-dimensional datasets. Deep learning techniques have been successfully applied in domains such as human action recognition [1–8], text processing and applications [9–14], medical image processing [15–26], and computational biology [27–36].

Conventional machine learning algorithms usually have some limitations in their ability to address natural data due to the complex structure of their raw form. For decades, constructing a classification model or pattern recognition system usually required careful investigation and considerable background information regarding the optimization problem to design a feature representation tool that transformed the input data (original data, such as the words of one review) into another data representation that is suitable for classifiers to process it and discover the classification pattern between the inputs and outputs. Feature reduction and feature learning are always important issues in the machine learning domain and are usually employed in combination with other machine learning algorithms, such as support vector machine, to provide a significant improvement in performance on recognition tasks compared to the standard machine learning algorithms. In addition, deep learning techniques can be seen as specific feature learning or feature reduction tools with multiple processing layers that abstract the high-level feature representation. A deep learning architecture usually contains one input layer, two or more processing layers, and one output layer. The degree of feature abstraction of the deep learning architecture is dependent on the depth of its neural network and the number of hidden neurons. However, although a more high-level network structure employed to construct a deep learning architecture may provide more powerful capabilities in feature representation, this is not suitable for only a few samples with low-dimensional attributes, and the choice of depth of neural networks is a trade-off between generalization capability and computational complexity.

In many forms of machine learning, shallow or deep, supervised machine learning is the most popular and frequently used method to construct a classification model or pattern recognition system. The main idea of supervised learning is to update the adjustable network parameters, i.e., weights and biases, by receiving a huge number of real-world samples, such as a car, a pet, a house or a person. To train a network using the supervised learning method, the first step is to collect a large volume of data from appropriate real-world domains, and each sample (instance) must be labeled with its category. During the training phase, these collected data are employed as input data for the classifier and then transformed into outputs through the many non-linear modules that connect the multiple processing layers of a deep network. Generally, we want an instance or a sample that is fed into a classifier to yield the desired output. However, this usually does not occur before training. Training a network requires modifying its adjustable parameters (weights and biases) so that the error between the predicted outputs and the desired outputs is as small as possible. The objective function, which is also called the loss function and is used to measure the training error, usually adopts the square loss measure for regression problems and the log-likelihood loss measure for classification problems. In a typical higher-level neural network using the supervised learning approach, there may be many processing layers with hundreds of millions of these tuning parameters (weights and biases), which are trained with hundreds of millions of samples with genuine labels.

Unsupervised learning is one type of machine learning method and can employ the “Deep Learn” concept to construct a higher-level neural network with more powerful capability for feature representation and abstraction. In many unsupervised learning methods, a deep belief network (DBN) is a more representative approach among these unsupervised learning. A DBN usually consists of many processing layers, and each processing layer contains multiple parameterized non-linear modules. DNB has a range of advantageous properties that can allow it to efficiently process large-scale and high-dimensional datasets. The main goal of a DBN is to learn a higher-level feature abstract representation that provides good classification performance. Actually, after network training is complete, the DBN eventually derives a set of vectors of parameters (optimal weights and biases) that are employed as initialization values of the network parameters of the multilayer perceptron to train a final model. These optimal parameters determined by the DBN, employed to initialize a deeper network, may result in a training network via which the model has more opportunities for finding the global optimal solution and avoiding the case in which the algorithm procedure becomes trapped in a local minimum. In addition to the original DBN algorithm, some improved algorithms have been developed [37–38]. The creation of an autoencoder is also a well-known unsupervised learning approach that can form a deeper network structure by stacking a group of independent autoencoders. Training a deep autoencoder is similar to a DBN in that a layer-by-layer search procedure is performed with hundreds of millions of unlabeled samples.

Using machine learning or deep learning approaches to solve a pattern recognition or time series prediction task usually requires constructing a more appropriate network structure based on the properties of the dataset and the data representation type. The network structure design requires considering the depth of the neural network and the number of hidden neurons. These network configurations are very influential factors on the performance of the network. In addition, training a deep learning architecture may be affected by the choice of hyperparameter configuration using the steepest gradient descent algorithm. Generally, proper adjustment of the weight depends on the gradient vectors calculated by the learning algorithm and the learning rate that controls the variation amplitude of the parameters. Therefore, selecting a more appropriate learning rate and other hyperparameters plays an important role in the network training phase and final constructed model. These hyperparameter configurations cannot be optimized by the steepest gradient descent algorithm. In addition, finding an optimal set of values for the hyperparameter configuration is a challenging task due to the large number of optimization variables and the complexity of the problem. However, in recent years, researchers have constructed a huge number of experiments to find various rules of thumb for the choice of hyperparameter configurations. These useful tricks can help improve the performance of deep learning approaches. For more details, see [39–42], which provide a variety of practical tricks for selecting the appropriate hyperparameter configuration. There are several other crucial hyperparameters that can produce effects on network training: dropout rate, momentum, decay, a*nd the number of* hidden neurons. In addition, weight initialization is always given by a randomly generated real-number vector that is small enough around the zero to yield the largest gradients during the early training phase. The learning rate is typically a hot topic and has been discussed in the machine learning community. The main reason is because it plays a more important role in network training compared to other hyperparameters. To find a more appropriate learning rate, there is a simple solution for choosing a fixed learning rate, that is, we can simply use a grid search approach in combination with a network training procedure using steepest gradient descent algorithms to determine an optimal learning rate from several candidate log-spaced values (10^−1^, 10^−2^, ….), based on the training error. In addition, a classical MLP classifier using the traditional steepest gradient descent algorithms, such as a back-propagation algorithm for training a deep learning architecture, has several limitations, i.e., the algorithm cannot find the global optimum solutions and its search procedure is easily trapped in local optima. Therefore, to solve these difficulties in using steepest gradient algorithms to train a higher-level neural network, the Stochastic Gradient Descent (SGD) algorithm has recently been proposed [43–48]. The SGD algorithm has several advanced advantages and therefore provides an efficient and practical solution for training a deep learning architecture. The properties of the SGD algorithm allow it only to optimize an objective function based on gradient information, and it is not able to process the hyperparameter configuration of deep neural networks. To solve the parameter estimation, many population-based stochastic search algorithms have been employed, including genetic algorithms (GAs) [49], particle swarm optimization (PSO) [50], differential evolution (DE) [51], fruit fly optimization (FOA) [52], and ant colony (AC) optimization [53]. Particle swarm optimization (PSO) is a typical swarm optimization algorithm and has shown impressive search performance for parameter optimization on a broad range of real-world applications. For example, Gaing Z L et al. [54] employed particle swarm optimization to determine the optimal proportional-integral-derivative (PID) controller parameters of an automatic voltage regulator (AVR) system. In this work, the PSO algorithm has been demonstrated as a more efficient and robust tool for improving the step response of an AVR system. Park J B et al. [55] presented a new approach based on the PSO algorithm for solving economic dispatch (ED) problems with nonsmooth cost functions; in this work, a modified PSO mechanism was proposed to address ED problems, and the experimental results demonstrated the superiority of the modified PSO algorithm compared to other evolutionary algorithms. Esmin A et al. [56] employed the PSO algorithm for a loss reduction study; in this work, the PSO algorithm was demonstrated to have promising results when applied to an IEEE-118-bus system. Ting T et al. [57] proposed a new hybrid particle swarm optimization (HPSO) for solving the unit commitment (UC) problem. The proposed hybrid algorithm used binary PSO and real-coded PSO to respectively process the UC problem and the economic load dispatch problem simultaneously. Ishaque K et al. [58] developed an improved maximum power point tracking (MPPT) approach based on a modified PSO algorithm for photovoltaic (PV) systems; this method can reduce the steady- state oscillation once the maximum power point (MPP) is located, which shows promising results compared with other existing methods. As shown by the above PSO-related work, the PSO algorithm has been successfully applied in a wide range of domains, such as the parameter estimation of control systems, economic problems, and other real-world applications. In this study, the PSO algorithm is presented as an ideal option for finding the hyperparameter configurations of deep learning architectures since its properties allow the particles to preserve the best previous experiences (important information regarding hyperparameter configurations) over generations. In this work, we propose an efficient approach using particle swarm optimization (PSO) in combination with the steepest gradient descent algorithm (gradient descent algorithm) to determine the optimal network structure and hyperparameter configuration before training the final model. The main contributions of this study are summarized as the five aspects below:

1. In this study, we propose an efficient approach that utilizes the advantages of the global and local exploration capabilities of the PSO algorithm and steepest gradient descent algorithm to automatically discover a more appropriate network structure with a better hyperparameter configuration for final neural network training.
2. In the proposed approach, the four crucial hyperparameters (learning rate, dropout rate, momentum, and weight decay) and the number of neurons of each hidden layers are considered for optimization. We design a simple parameter representation that encodes the network configuration (network structure and hyperparameters) as a real-number vector as the individuals of PSO in the search process such that real numbers can be efficiently processed.
3. The proposed approach can provide a flexible method to construct an ensemble model and a well-performing DNN classifier by using the final solutions of the PSO algorithm to initialize DNN classifiers and train them with their corresponding hyperparameter configurations on the entire training data. Specifically, the local best (*pbest*) and global best (*gbest*) solutions of the PSO algorithm are employed to construct the ensemble model and the individual DNN classifier, which maximize the generalization capability and efficiency, respectively. In addition, the flexibility of the proposed approach allows for any number of classifiers to be combined to form an ensemble based on their scores, which are the last training accuracies during the training phase. This process directly chooses a certain number of DNN classifiers with the highest scores from the candidate classifiers trained by the network configurations expressed by the solutions (*pbest*) of the PSO algorithm without training any new DNN classifiers.
4. In this study, we have evaluated the performance of the ensemble model and the individual DNN classifier that are generated by the proposed approach. The empirical results demonstrate that the proposed approach of using PSO in combination with steepest gradient descent algorithms can maximize their local and global exploration capabilities and find optimal solutions that lead to better performance for both network training and the final models.
5. This study has investigated the influence of various ensemble models with different numbers of classifiers and depths of the neural networks.

The rest of this paper is organized as follows. A brief introduction to artificial neural networks is presented in section 2. A detailed description of our proposed approach using PSO in combination with steepest gradient algorithms to optimize deep learning architectures is presented in section 3. The detailed experimental results of using the proposed approach with various parameter configurations are reported in section 4, and we also investigate the effects of the ensemble model with different network depths and different numbers of combined classifiers. Finally, the conclusions are illustrated in section 5, and we also discuss future directions.

## 2. Background materials

In this section, we provide the details of the deep learning architectures and hyperparameter configuration. The contents of this section are organized as follows. Subsection 2.1 presents a brief overview of deep learning architectures. Subsection 2.2 describes the details of the Stochastic Gradient Descent (SGD) algorithm and network training.

### 2.1 A brief overview of deep learning architectures

In this subsection, we will describe the MLP classifier in detail. MLP is one of the most popular and classical machine learning approaches and is inspired by the neurotransmissions of the human brain. MLP can be regarded as a combined model associated with an artificial neural network (ANN) with many hidden layers and neurons, which has been successfully applied in computer vision and other real-world applications. A classical ANN classifier is generally comprised of three connected layers (one input layer, one hidden layer, and one output layer). The number of neurons of the input layer is fixed according to the size of the actual input data, and the number of neurons in the output layer matches the size of the actual outputs. Many hidden layers with hidden neurons can construct a flexible neural network, and a more complicated network with a huge number of hidden layers and many neurons usually requires a huge number of training samples and more computational energy and time for training. When a new sample is fed into a network, the input layer first receives the original data and makes a sum of activations with respect to a hidden neuron; the sum is then converted to a hidden neuron’s output activation by a nonlinear function, which is defined as follows:

$$\begin{array}{c} O=\left(I_{i}\cdot W_{ij}+b_{j}\right)\\ f_{j}\left(I_{i}\right)=\frac{e^{o}-e^{-o}}{e^{o}+e^{-o}} \end{array}$$ (1)

In Eq (1), where *o* denotes the sum of the input data with respect to the weights and biases of the j-th neuron, *f*_*j*_ (.) is the hyperbolic tangent function that calculates the activation value of the j-th neuron, *I*_*i*_ = (*I*_1_, *I*_2_, …, *I*_n_) is the input data of a single sample, and *W*_*ij*_ = (*W*_1*j*_, *W*_2*j*_, …, *W*_*nj*_) is a weight vector of the j-th neuron of the hidden layer. A simple MLP learning architecture generally consists of three connected layers, including one input layer, one or more hidden layers, and one output layer. To address the regression problem using the MLP classifier, the most popular performance criterion (also called cost) is the mean square error (MSE), which is defined as follows:

$$MSE\left(Y,Y^{\prime }\right)=\frac{1}{n}\sum_{i=1}^{n}\left(Y_{i}-Y_{i}^{\prime }\right)\cdot \left(Y_{i}-Y_{i}^{\prime }\right)$$ (2)

where *Y* and $Y^{‘}$ denote the actual output value and the predicted output value, respectively, and *n* denotes the number of instances. To train an MLP classifier, the frequently used learning technique is the back-propagation algorithm, which dynamically updates the parameters (weights and biases) in the direction that the gradient descent aims to find the optimal parameters. To address a classification problem using an MLP classifier, the common performance criterion is the mean squared error, which is shown as follows:

$$E=\frac{1}{2}\sum_{i=1}^{n}\left(Y_{i}-Y_{i}^{\prime }\right)\cdot \left(Y_{i}-Y_{i}^{\prime }\right)$$ (3)

where *E* is the cost measure between the actual labels and the outputs, which is employed to calculate the gradient; *Y*_*i*_ and $Y_{i}^{‘}$ denote the actual label and the predicted label of the i-th instance, respectively; and *n* is the number of instances.

### 2.2 A brief overview of SGD and network training

In most cases, the learning architectures used in training are mostly used to solve classification problems. During the training phase with the supervised learning method, a DNN classifier generally requires an objective function to evaluate the training error. The common function is zero-one loss, which minimizes the number of errors on training samples. It has a simple form: $g_{loss}=\sum_{i=0}^{n}I\left(f\left(x^{i}\right)\neq y^{i}\right)$, where *I* is ‘1’ if *f*(*x*^*i*^) ≠ *y*^*i*^ and ‘0’ otherwise. Because the zero-one loss function is not differentiable, optimizing it is prohibitively expensive when a large model with a huge number of parameters (weights and biases) is being trained. Therefore, the original formula can use the log-likelihood form as follows: $g_{loss}=\sum_{i=0}^{n}\text{log}P\left(Y\neq y^{i}|x^{i},\theta \right)$. Generally, we aim to solve the minimization of a loss function instead of maximization. We use the negative log-likelihood as a loss function in the training of a deep learning architecture. The negative log-likelihood function in the classifier is differentiable. This means that the gradient of the loss function over the training data can be used as a signal in supervised learning of a DNN classifier. During the training phase, some gradient descent algorithms are employed to adjust the parameters by making small steps to minimize the error of a loss function. The ordinary gradient descent algorithm generally has a simple form, but it usually provides significant performance when training a neural network. The stochastic gradient descent (SGD) algorithm is similar to the principles of the original gradient descent, but it gains more benefits by calculating the gradient from just a few samples at a time instead of the entire training data. In addition, the variant in the stochastic gradient descent algorithm is the adoption of the “minibatches” concept. SGD using the mini-batch method is similar to the original SGD. The difference is that the mini-batch technique used in SGD can help to reduce the variance in the estimate of the gradient and can work better in the hierarchical memory organization of powerful computers. In addition, when we train a deep neural network, the training processing may overfit the training data. The regularization concept is used to combat overfitting, and server techniques have been proposed. L1 and L2 regularization are common approaches that add an extra term in the loss function for the purpose of penalizing certain parameter configurations. The other approach is called “Early stopping,” which is employed to address overfitting by observing the classifier’s performance on an independent validation set.

## 3. Methodology

**Fig 1:**
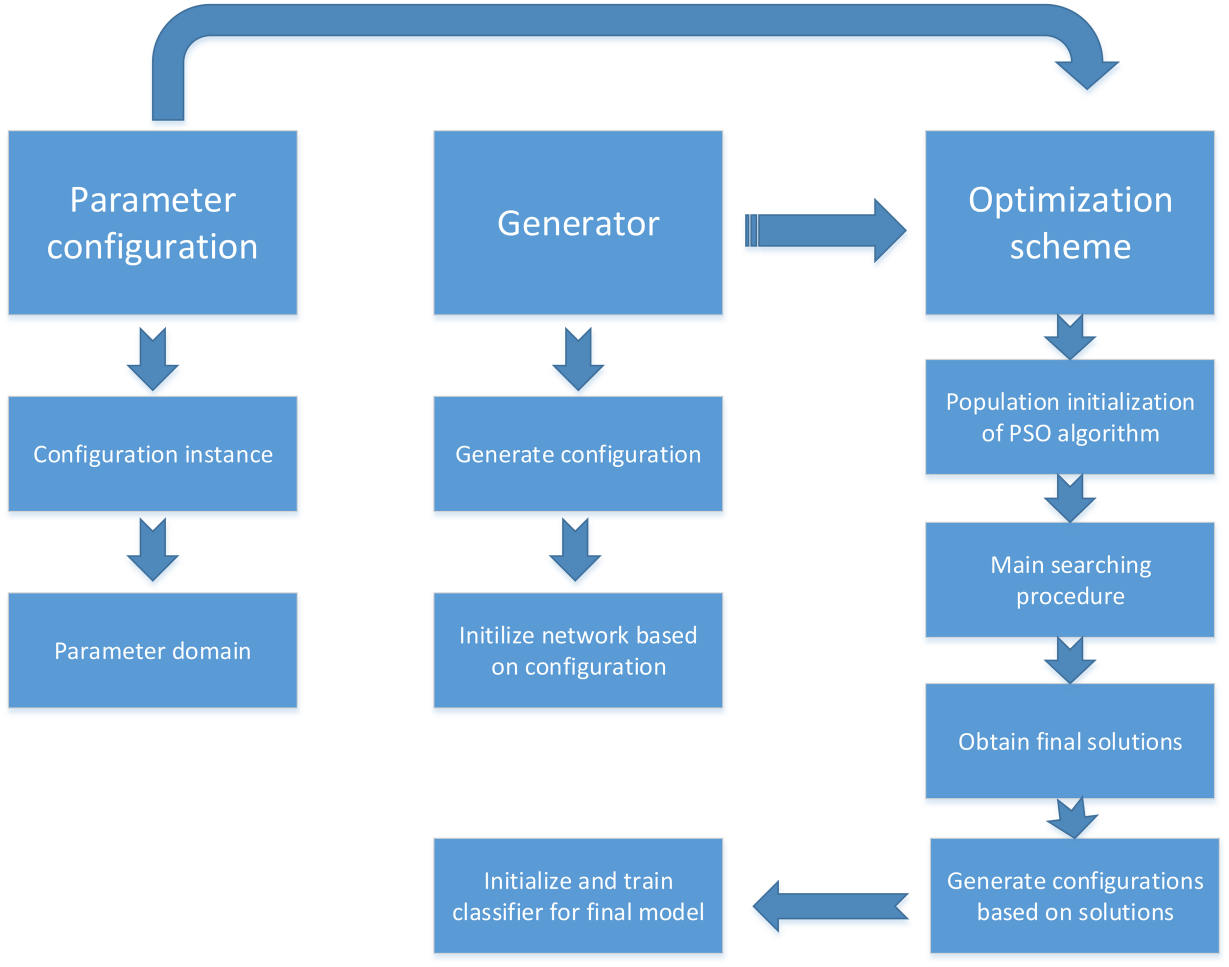
The basic framework of the proposed approach.

In this study, we present a new deep learning approach that automatically determines one or better hyperparameter configurations of the neural network to further improve the classification performance and generalization capabilities on various pattern recognition and regression tasks. Because network structure (different numbers of processing layers and neurons) and hyperparameter configuration play important roles in the training phase of deep learning architectures, various network structures and hyperparameter configurations employed to train a network may result in the derivation of a set of models that generally have different performances on pattern recognition tasks. Finding an appropriate network structure and hyperparameter configuration for a deep learning architecture is a difficult challenge due to the complexity of deeper networks and the high-dimensional optimization parameters. In addition, an individual classifier (network) employed to predict results on a large-scale and high-dimensional dataset has several limitations, including weak generalization ability and instability in the training phase. To solve the above issues and further improve the performance of deep learning architectures in pattern recognition and regression tasks, the proposed approach mainly aims to construct a robust and efficient model by using a more appropriate network structure and optimal hyperparameter configuration determined by an efficient optimization scheme using particle swarm optimization (PSO) and steepest gradient descent algorithms. During the optimization scheme, the advantages of the global and local search capabilities of PSO and steepest gradient descent algorithms can provide a powerful and efficient search process for finding the best network structure and hyperparameter configuration. Fig 1 shows the basic framework of the proposed approach. As shown in the graph, the framework consists of three independent modules. The first module is called the basic element model and defines the various network structures and optimization parameters with their search domains. The second module is called the generator and is mainly responsible for generating various deep learning architectures depending on different network configurations (numbers of processing layers and neurons and network type). The third module is called the optimization scheme and implements the cyclic process to determine the final model by using PSO in combination with steepest gradient descent algorithms determining the best solution (best network structure and hyperparameter configuration). The remainder of this section is organized as follows. Subsection 3.1 presents the detailed basic element model and generator. Subsection 3.2 provides a brief overview of the PSO algorithm, and we also provide a coding design for hyperparameter representation. Subsection 3.3 describes the details of the ensemble model with multiple combined classifiers. Subsection 3.4 presents an efficient and robust optimization scheme using PSO and steepest gradient descent algorithms.

### 3.1 The basic element model and generator

The basic network configuration comprises a neural network structure and a hyperparameter configuration. In the proposed approach, the basic deep learning architecture adopts the classical multilayer perception (MLP), which generally consists of one input layer, two or more processing layers, and one output layer, where neurons in the input layers are equal to the size of the input features, neurons in the processing layers generally can be any number, and neurons in the output layer are equal to the number of categories for the classification model and equal to 1 for the regression model. The proposed approach can provide a flexible way to initialize the network for classification and regression problems. In addition, the prediction performance of the generated deep learning architecture extremely depends on the network training using the hyperparameter configuration. The choice of hyperparameter configuration directly influences the performance of the steepest gradient descent algorithm in the training phase of the neural network, which is a very important issue in our study. The difference between training a deep learning architecture and a shallow learning architecture is that a deep learning architecture requires the initialization of more hyperparameters before training. The neural network structure and hyperparameter configuration are usually not fixed because these configurations depend on the properties of the datasets. We can select more hyperparameters as optimization parameters to be searched using optimization algorithms to derive a well-generalized model, but the complexity of the parameter searching process is considerable. Selection of a few hyperparameters as optimization parameters may result in the derivation of a model that only yields slight improvements in performance compared to the normal method. Therefore, the proposed approach only selects several crucial hyperparameters as optimization parameters due to their impacts on network training. These important optimization parameters are learning rate, dropout rate, decay, momentum, and the numbers of processing layers and neurons, which play an important role in the training phase. In addition, searching for these optimization parameters employed to construct a deep learning architecture requires an appropriate search range for each optimization parameter, which aims to avoid the occurrence of wrong numbers and ensure that each searched parameter is always reasonable and correct. Moreover, the search ranges for optimization parameters can also help to reduce the computational time of the search procedure because we can set a small search range for each optimization parameter so that the PSO algorithm only requires a few search iterations to determine the optimal solution. The values of these search ranges of optimization parameters are dependent on the properties of the dataset (the size of input features and number of training samples) and optimization tasks (classification or regression problems).

The generator is employed to generate a network configuration containing a network structure and a hyperparameter configuration in a random manner. In this generated model, each of its hyperparameters is given by a random value corresponding to the parameter domain.

### 3.2 A brief overview of PSO and its coding design for hyperparameter representation

In this section, we present a brief overview of the particle swarm optimization algorithm and a simple coding design for a representation of the network structure and hyperparameter configuration. In recent years, a variety of population-based intelligent algorithms inspired by biological mechanisms have been developed to solve a variety of complex problems and successfully applied in real application tasks, including medicine, engineering, computer science, and finance problems. Among most intelligent algorithms, swarm intelligence can be considered one type of artificial intelligence concept or technique that was inspired by the natural phenomenon of a flock of birds searching for food sources by changing their locations based on their former position and swarm position. Particle swarm optimization (PSO) is one of these swarm techniques and was first introduced by Kennedy J, Eberhart R in 1995 [1][59]. Particle swarm optimization is similar to other population-based meta-heuristic optimization techniques in that it first initializes a group of individuals as a population and then updates the information (state) of these individuals by an evolution process. The advantages of the PSO algorithm compared with other swarm intelligence algorithms is that the PSO algorithm generally contains a simple and efficient search process, is easy to implement, and can efficiently find global optimal solutions that are closest to the actual solutions.

Particle swarm optimization employs particles as population members, and each particle (individual) is expressed by an m-dimensional real-number vector. During the evolution process of the PSO algorithm, each particle of a particle swarm (population) is considered to be a representation of a possible solution in a finite search space (m-dimensional search space). PSO first initializes a group of particles as a population in a random manner. After the initialization of the PSO population, an evolution procedure is performed with a certain number of generations, and during each generation, each particle (individual) finds a possible optimal solution by changing its direction depending on the two crucial factors of position and velocity of the individual best previous experience (*pbest*) and the best previous experience of all individuals/swarm particles (*gbest*). The details of the velocity and position update of an individual can be seen in Eqs (4) and (5), where *t* and *t* + 1 denote the generations (iterations), *d* denotes the number of dimensions of the particle, *X*^*t*^(*i*) denotes the position in the i-th dimension of the particle at generation *t*, and *V*^*t*^(*i*) denotes the velocity of the i-th dimension of the particle at generation *t* + 1. *R*_1_ and *R*_2_ are randomly generated values in the domain of [0, 1]. *W* denotes an inertia weight that was first proposed by Shi and Eberhart [1][57]. *C*_1_ and *C*_2_ are positive acceleration coefficients, which are also called cognitive and social parameters due to their role in the algorithm evolution procedure. In fact, these two important parameters are mainly employed to control the balance of an individual’s self-learning versus learning from the entire PSO population.

$$V^{t}\left(i\right)=W\cdot V^{t+1}\left(i\right)+C_{1}\cdot R_{1}\cdot \left(pbest^{t}\left(i\right)-X^{t}\left(i\right)\right)+C_{2}\cdot R_{2}\cdot \left(gbest^{t}\left(i\right)-X^{t}\left(i\right)\right),i=1,2,‥,m$$ (4)

$$X^{t+1}\left(i\right)=pbest^{t}\left(i\right)+V^{t+1}\left(i\right),i=1,2,\ldots .m$$ (5)

To further improve and balance the relationship between the local exploitation and global exploitation, we use the time-varying acceleration coefficients (TVAC) [60, 61] and time-varying inertial weight (TVIW) [60, 61, 62]; the effectiveness of using TVAC and TVIW techniques on the acceleration coefficients and inertial weight have been verified. These two approaches dynamically update the acceleration coefficients and inertial weight during the iterations and can help the original PSO algorithm perform better in determining the region of the global solution and avoiding the case of the algorithm search procedure becoming trapped in local minima [60, 61, 62].

When using the TVAC approach, the acceleration coefficients *C*_1_ and *C*_2_ are adjusted based on the initial values of the acceleration coefficients *C*_1*i*_ and *C*_1*f*_ and the current iteration. The details of the acceleration coefficient update process are shown in Eq (6), where *t* and *t*_max_ denote the current generation (iteration) and the maximum number of generations, respectively. In addition, the TVIW approach is employed to change the inertial weight during the evolution process. The details of the inertial weight *W* update are seen in Eq (7), where *W*_max_ and *W*_min_ denote the maximum and minimum values of the inertial weight. The TVIW approach can efficiently balance the global exploitation and local exploitation of the PSO algorithm, that is, a large inertial weight *W* may allow the PSO algorithm to exhibit better global search capability at the beginning of the algorithm procedure, and the local search ability of PSO algorithm is gradually increased by gradually decreasing the inertial weight *W* in a linear manner during the algorithm evolution procedure.

$$C_{\text{1}}=C_{\text{1i}}+\frac{t}{t_{\text{max}}}\left(C_{1f}-C_{1i}\right)$$ (6)

$$C_{\text{2}}\text{=}C_{\text{2i}}+\frac{t}{t_{\text{max}}}\left(C_{\text{2}f}-C_{\text{2}i}\right)$$ (7)

$$W=W_{\text{max}}-\frac{t}{t_{\text{max}}}\left(W_{\text{max}}-W_{\text{min}}\right)$$ (8)

From these above equations, the initial values of the inertial weight *W*_max_ and *W*_min_ *W*_max_ are usually constant values. The initial values of the acceleration coefficients *C*_1*i*_, *C*_1*f*_, *C*_2*i*_, *C*_2*f*_ are set to constant values.

### 3.3 Combining the evidence of multiple classifiers

In this subsection, we present an efficient and configurable ensemble model whose members (sub-classifiers) can be constructed by any deep learning architecture. Because combining the evidence of multiple DNN classifiers may provide better generalization performance than an individual DNN classifier but requires more computational times to implement all of the training processes, we propose an efficient and flexible approach to build an ensemble model that aims to minimize the model complexity and does not deteriorate the generalization capability. To construct such an ensemble model, we directly choose a certain number of DNN classifiers with the best scores (training accuracies calculated on an independent validation dataset using the trained DNN classifiers) from the entire set of DNN classifiers that are initialized and trained by the final solutions (*pbest*) of the PSO algorithm without training any new DNN classifiers. Let *C* = {*C*_1_, *C*_2_, *C*_3_, ….} be a set of DNN classifiers that were trained with the optimal hyperparameter configurations using the steepest gradient descent algorithm on the entire training dataset. Let *S* = {s_1_, *s*_2_, *s*_3_, ….} be a set of scores corresponding to the set of DNN classifiers *C*. Let *E* = {*e*_1_, *e*_2_, *e*_3_, …, *e*_*h*_} be a subset of *C* and its members be selected based on their scores. *h* is a threshold value employed to control the number of members of an ensemble model. After an ensemble is generated, a fusion function with a majority vote rule is employed to calculate a final output. The detailed calculation is presented as follows. Let $O=\left\{o_{1}^{i},o_{2}^{i},o_{3}^{i},\ldots ,o_{c}^{i}\right\}$ be a binary decoded for an output of the i-th sample of a DNN classifier; then, the classifier was trained with ($o_{t}^{i}=1$ and $o_{j}^{i}=0$ for *j* ≠ *t*, 1 ≤ *j* ≤ *a*) for an observation of class *t*, where *a* denotes the number of categories. During the prediction phase, an observation will be classified as category *t* when $o_{t}^{i}>o_{j}^{i}$, for all *j* ≠ *t*, 1 ≤ *j* ≤ *a*. To predict an output using the ensemble model, the combination of evidence over all classifiers uses a fusion function with a majority vote rule that calculates the outputs based on the decision made by most of the members. Then, we defined a function $f_{j}=\text{max}\left\{o_{1}^{j},o_{2}^{j},\ldots .,o_{a}^{j}\right\}$ to calculate an output of the j-th classifier. An observation will be classified into category *t* when *f*_*t*_ > *f*_*j*_, for all *j* ≠ *t* and 1 ≤ *j* ≤ *a*. Combining evidence from multiple DNN classifiers would generally result in construction of a well-performing model for which some misclassified observations are ignored by most correctly labeled observations.

### 3.4 The optimization scheme using PSO and steepest gradient descent algorithms

**Fig 2:**
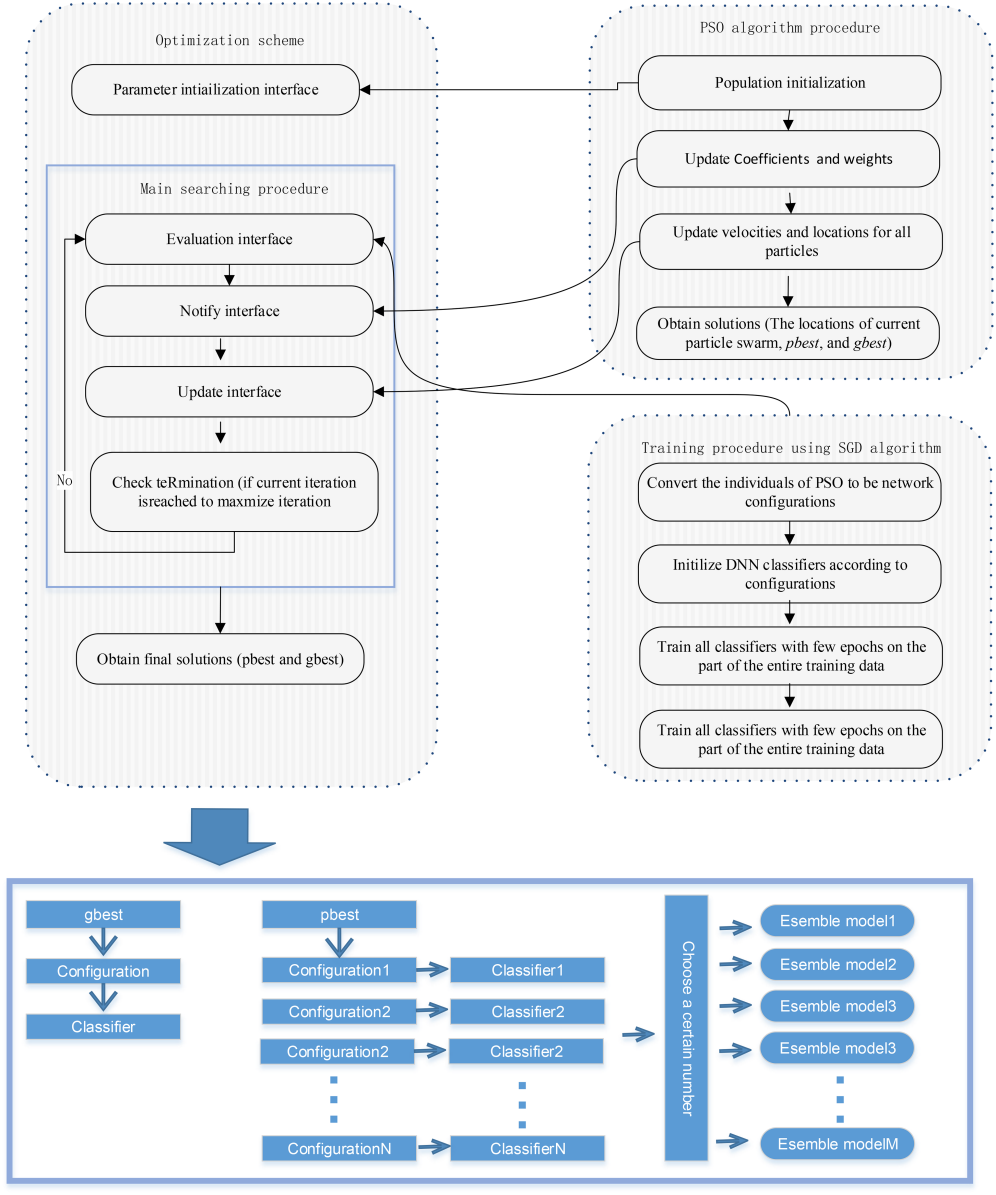
The basic framework of the optimization scheme using PSO in combination with stochastic gradient descent algorithms.

**Fig 3:**
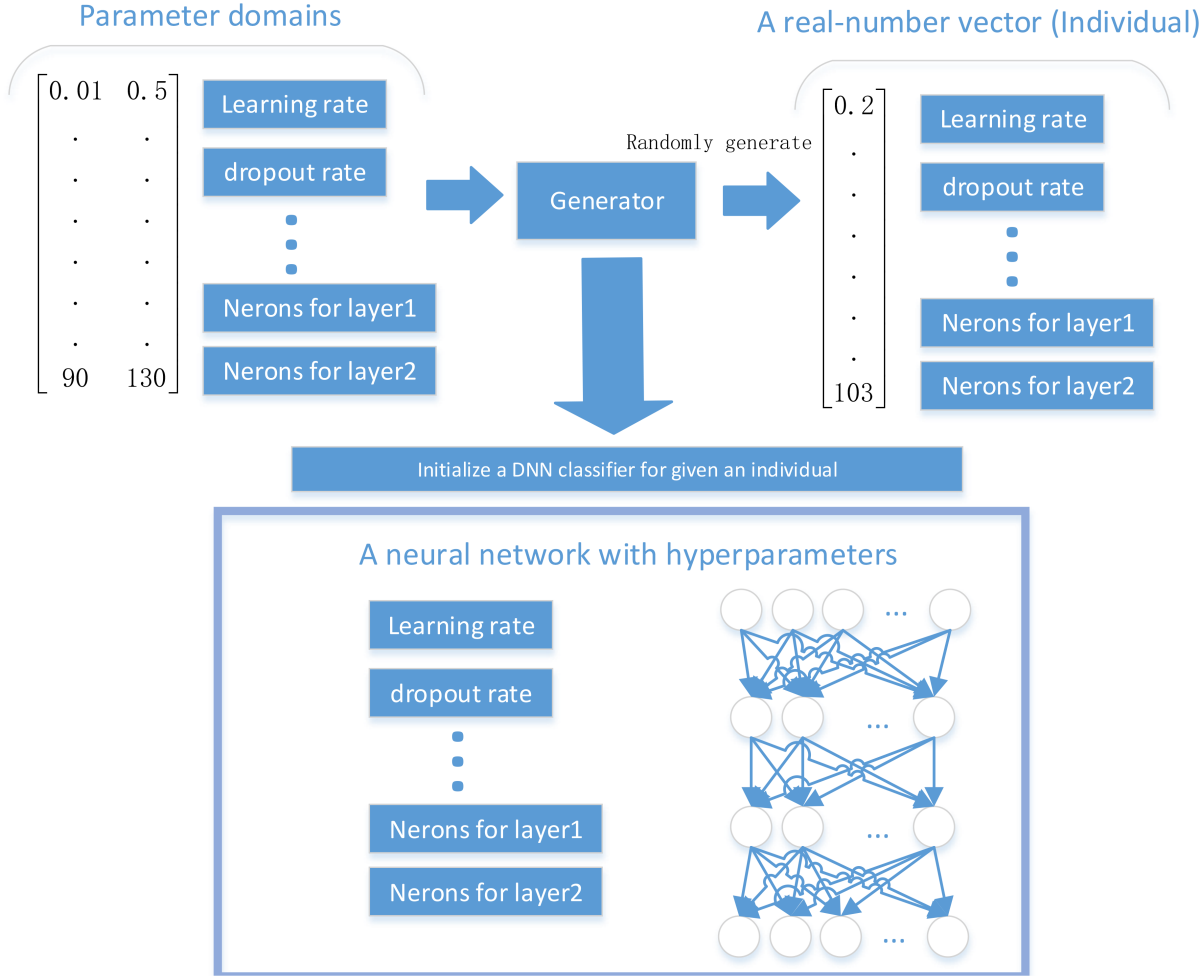
The parameter representation design for the individuals of the PSO algorithm.

In the previous subsections, we have described the details of the search procedure and coding design of the hyperparameter configuration of the PSO algorithm. In this subsection, we present an efficient and robust optimization scheme based on a hybrid search approach using PSO and steepest gradient decent algorithms. The optimization goal of the proposed scheme is to automatically determine one or better network configurations (network structures and hyperparameter configurations) before using the deep learning architecture in applications. Because the performance and generalization capability of a deep learning architecture extremely depend on the network structure and hyperparameter configuration during the training phase, the choices of network structures and hyperparameter configurations employed to train a deeper network play an important role in our proposed approach. In addition, our proposed approach provides a configurable and flexible method for implementing a parameter-searching process. Any optimization algorithms can be fitted in these interfaces to implement their population initialization, population evaluation, and location updating. Fig 2 displays the details of this parameter-searching process, and as shown in the graph, the entire search process is comprised of 3 independent interfaces. These implemented interfaces are the parameter initialization interface responsible for population or parameter initialization of algorithms; the update interface provides a notice for the state or location update of algorithms after the evaluation interface has been utilized; the evaluation interface is mainly responsible for calculating the scores of multiple network configurations. In this manner, the particle swarm (individuals) of the PSO algorithm can convert their information to network configurations to obtain the scores that are employed to evaluate the population; finally, these operating interfaces are designed to correspond to the primary procedure of the optimization algorithm, and a main search process is performed in which these operating interfaces are performed in a sequence until termination of the procedure. Therefore, we can use other advanced population-based stochastic search algorithms to implement this optimization algorithm. The main reasons for using the PSO algorithm without other optimization algorithms are its powerful search capability for determining the global optimum and its convenient representation of continuous variables. Furthermore, the advantages of the global and local search capabilities can be explored by a hybrid approach using PSO in combination with steepest gradient descent algorithms. The main idea of the hybrid approach is that the PSO algorithm is employed to search for a neural network and hyperparameter configuration by adjusting the locations of the particle swarm, and then each particle (individual) representing a prototype is converted into a network configuration to initialize a classifier after performing a training procedure with a small step of mini-batch learning using the steepest gradient descent algorithm. After the network training, the last training loss value or training accuracy, depending on the independent validation set, is employed as the score (fitness value) for the individual. The basic process of the optimization scheme using PSO and the steepest gradient descent algorithm is summarized by five independent steps as follows:

(1) Initializing parameters for PSO and the optimization scheme:
  Similar to the other population-based stochastic search algorithms, the PSO algorithm requires initialization of the population size, the maximum number of iterations, the acceleration coefficients *C*_1*i*_, *C*_1*f*_, *C*_2*i*_, *C*_2f_, and the inertial weight *W*_min_, *W*_max_. To initialize parameters for the optimization scheme, the domains of the optimization parameters must be initialized. These optimization parameters are learning rate, dropout rate, momentum, decay, and the numbers of neurons of all processing layers. The lower and upper bounds are used to define the domain for each optimization parameter, which can ensure that each optimization parameter is searched for in its corresponding search range. In addition, the epochs for the validation phase and final training phase usually set small and large values to reduce the computational time spent evaluating the PSO individuals (particle swarm) representing classifiers and ensure that the deep learning architecture fits the training samples before using them for any pattern recognition tasks. The next step is population initialization of the PSO algorithm.
(2) Population initialization of the PSO algorithm:
  In the proposed optimization scheme, each particle (individual) of the PSO algorithm is used to represent a deep learning architecture with a hyperparameter configuration. To initialize the population of the PSO algorithm, each dimension of a particle denotes an individual optimization parameter of the network configuration and is generated by a random real number in its corresponding parameter domain. After the population is initialized, we can obtain a set of candidate network structures and their corresponding hyperparameter configurations, which are expressed by population. Therefore, we can evaluate the population of the PSO algorithm by training these deep learning architectures with their corresponding hyperparameters and then validating them on the independent validation set. The details of these training and validating processes are presented as follows:
(3) Population evaluation using the steepest gradient descent algorithm:
  This step is mainly responsible for evaluating the population of the PSO algorithm by training and validating the neural networks using the steepest gradient descent algorithm. In the evaluation phase, each particle (individual) can be viewed as a representation of a potential solution and thus is transformed into a network configuration. Then, the generator initializes a deep neural network according to the network configuration. After all the neural networks are initialized, these DNN classifiers are then trained using the steepest gradient descent algorithm with a few steps of mini-batch learning processes on an independent training subset that is randomly collected from the entire training dataset according to a predefined threshold value. After all DNN classifiers are trained, the scores of the individuals are calculated by evaluating these trained DNN classifiers by predicting the output on an independent validation dataset. The details of the training and validating processes using the steepest gradient descent algorithm are displayed in Fig 3 and can be described as follows. Let *D* denote the entire training dataset and be randomly divided into two independent sets *D* = {*Tr*, *Te*} by a threshold *λ*, where *Tr* and *Te* denote the independent training and validation sets, respectively, and *λ* is employed to control the size of the training set and is usually set to 0.8. Let *P* = (*P*_1_, *P*_2_, ……, *P*_*n*_) denote the population of the PSO algorithm and *P*_*i*_ = (*C*_1_, *C*_2_, ……, *C*_*m*_) denote the i-th particle (individual) consisting of *m* optimization parameters. After initializing the population, each particle *P*_*i*_ is employed to construct a deep learning architecture and its corresponding hyperparameter configuration, which are denoted as *P*_*i*_ = {*Net*, *C*}, where *Net* and *C* denote the deep learning architecture and hyperparameter configuration, respectively. Then, the deep learning architecture *Net* is trained with the mini-batch learning method using the training parameters *C* on the independent training set *Tr* several times. After network training, we can calculate the training accuracy based on the independent set *Te* or the last loss value after network training as a score (fitness value) for the individual *P*_*i*_ of the PSO algorithm. After calculating the scores for individuals, the local best experience (score and location) of the individual can be determined by comparing the current score of the individual *P*_*i*_ and the best score from its previous experiences in past generations and replacing the previous local best experience by the current best if the current score is large than the previous local best score. Similar to the local best experience, the global best experience is determined by comparing the current score of the individual *P*_*i*_ and the best score from the previous experiences of all individuals in past generations and replacing the previous global best experience with the current best if the current score is large than the previous global best score. After determining the local and global best experiences (scores and locations), Eqs (2) and (3) are employed to update the velocities and locations of the particle swarm of the PSO algorithm. The next step is checking the algorithm termination as follows:
(4) Checking algorithm termination:
  This step is mainly responsible for checking the algorithm termination (if the number of iterations has reached the maximum iteration). If the condition is satisfied, then the algorithm stops and we go to step (6); otherwise, we continue to step (5).
(5) Training a final model and evaluation:
  After the algorithm search procedure, we can obtain a set of local solutions (*pbest*) and the global best solution (*gbest*), and we then use them to initialize a set of optimal classifiers and an individual classifier, respectively. Then, each classifier is trained by a training procedure with a certain number of mini-batch learning iterations on all of the training samples. During the training phase, the classifier has a corresponding hyperparameter configuration that is employed in the steepest gradient descent algorithm to adjust the network parameters (weights and biases) to determine the best solution that allows the training error or loss value between the input pattern and output pattern to be as small as possible. After all classifiers have been trained, we can obtain an individual trained classifier that is initialized by the global best solution (*gbest*) of the PSO algorithm and a set of optimal trained classifiers that are initialized by the global best solution of the PSO algorithm. In the performance evaluation phase, we evaluate the performance of the proposed approach by predicting all testing samples using a combined model (ensemble model) of multi-classifiers with a majority vote strategy and an individual classifier. In addition, the proposed approach provides a flexible way to construct an ensemble model for a given number. The main idea of using a certain number of classifiers to form an ensemble model in our approach is to directly select classifiers from the generated classifiers that have been initialized by the local best solutions (*pbest*) and trained on all of the training samples, without training new classifiers. As a result, a light ensemble model with a small number of classifiers can be constructed by selecting a few optimal classifiers whose final training loss values or training accuracies, depending on the independent validation set, are superior to the remaining classifiers. In this manner, a light ensemble model can reduce the computational time in predicting a huge number of samples and may maintain the original classification performance and generalization ability. In the following experiments, we have also performed several tests using these ensemble models with different numbers of sub-classifiers to investigate the relationship between the performance metrics (prediction and generalization performance) and the number of classifiers.

## 4. Experimental studies

In this section, we constructed several experiments to evaluate the performance of the proposed approach and investigate the influences on performance when changing the deep learning architecture and hyperparameter configuration. More specific experimental contents are organized as follows. The details of the experimental dataset, experimental setting, and data pretreatment are described in subsection 4.1. The experimental results for a classification problem using the MNIST dataset are reported in subsection 4.2. The experimental results for a regression problem using the biological activity datasets are reported in subsection 4.3. The investigation of the influence of the use of a deeper network structure is presented in subsection 4.4. The influences of the use of various PSO algorithm parameter configurations are investigated in subsection 4.5.

### 4.1 The datasets, experimental setting, and data pretreatment

In this subsection, we provide the details of the datasets, experimental setting, and data pretreatment. The proposed approach mainly solves classification and regression problems. To evaluate the performance of the proposed approach on a classification problem, the MNIST dataset [63] is used. The MNIST dataset is the most popular and frequently used dataset employed to evaluate the performance of various machine-learning algorithms. The main goal of the use of the MNIST dataset is to recognize the handwritten characters from the digital images. The MNIST dataset is also a well-known standard benchmark used to evaluate the performance of machine learning approaches. Each digital image of the MNIST dataset is expressed by a real-number matrix comprised of image pixels (28 rows and 2 columns) varying from 0 to 255. The numbers of training samples and testing samples are 60000 and 10000, respectively. To evaluate the performance of the proposed approach on a regression problem, the kaggle competition dataset (KCD) is used. The KCD dataset is downloadable from www.kaggle.com and was held by merck sponsor (2012). To solve the regression problem in this study, all 15 targets of the KCD datasets are chosen as experimental datasets. Each target of the KCD datasets contains many numbers of molecules (samples) and descriptors (features), and their values are real numbers. The main goal of the use of the KCD datasets is to predict the biological activity values based on the descriptors. More details of the KCD datasets are illustrated in Table 1. The proposed approach is implemented based on the keras and theano libraries using Python 2.72. All experiments are performed on a personal computer with an Intel Core i5 CPU running at 2.53 GHZ, 8 GB of RAM, and the Windows 10 operating system. The experimental environment is constructed in 64-bit Python, version 2.72. The computational times in the training and testing phases are recorded for further analysis. Before any processing of image datasets, to improve the classification accuracy and convenience of numerical calculation, the data pretreatment is used to scale the data into the interval [0, 1] or [−1, 1]. In general, we can obtain a processed image for which each feature (pixel) is scaled into the interval [0, 1] by dividing the feature value by the constant 255.

**Table 1:** The details of fifteen different biological activity datasets.

Index of the dataset	Dataset name	Number of molecules	Number of descriptors
1	3A4	50000	9491
2	CB1	11640	5877
3	DPP4	8277	5203
4	HIVINT	2421	4306
5	HIVPROT	4311	6274
6	LOGO	50000	8921
7	METAB	2002	4595
8	NK1	13482	5803
9	OX1	7135	4730
10	OX2	14875	5790
11	PGP	8603	5135
12	PPB	11622	5470
13	BAT_F	7621	5698
14	TDI	5559	5945
15	THROMBIN	6924	5552

### 4.2 Results and analysis for the MNIST dataset

In this section, we evaluate the classification performance and generalization ability of the proposed approach in solving the recognition of handwritten characters. In this experiment, two different methods were used to construct the final classification models. One approach is called “DNN-NONPSO” and randomly generates a group of network configurations (network structures and hyperparameter configurations) from the domains of the parameters and uses them to directly train a set of final deep learning architectures with their corresponding hyperparameter configurations on all of the training samples. The second approach is called “DNN-PSO” and first implements an optimization scheme in which the network configuration is decoded into a real-number vector and employed as a particle (individual) of the PSO algorithm so that the algorithm search procedure can efficiently process these optimization parameters. In addition, after once updating the particle swarm information, a particle (individual) of the PSO algorithm is converted into a network configuration and employed to initialize a deep learning architecture and train the network with a few steps of mini-batch learning to obtain the final training loss value as the score of the individual. Finally, the determined optimum solutions are employed to construct the final models. The detailed parameter configurations for the PSO algorithm and optimization scheme are presented as follows. The population size is set to 20, and the maximum number of iterations of PSO is set to 30. The acceleration coefficients *C*_1*i*_, *C*_1*f*_, *C*_2*i*_, *C*_2f_, are set to 2.5, 0.5, 0.5, and 2.5, respectively, and the inertial weights *W*_min_, *W*_max_ are set to 0.4 and 0.9, respectively, according to the recommendations. The domains of the network structure and hyperparameter configuration are set as follows. The search ranges of the learning rate, decay, momentum, and dropout rate are set to [0.01 0.9], [0.0001 0.0001], [0.1 0.9], and [0.1 0.9], respectively. Due to only two processing layers being employed to construct the deep learning architecture in the first experiment, the search ranges of the numbers of neurons of the two processing layers are set to the same range: [90, 150]. During the network training phase, the epochs for population evaluation of the PSO algorithm and training of the final model are set to a small number and a large number of epochs (5 and 20), respectively. A batch size of 100 is set for network training using mini-batch learning.

To evaluate the performance of the proposed approach on the MNIST dataset, the k-fold cross-validation technique is used. The k-fold cross-validation technique is the most popular and frequently used method and is mainly employed to evaluate the performance of different algorithms in an unbiased manner. The main purpose of using k-fold cross-validation in this experiment is that the k-fold cross-validation technique has been widely applied in most studies and hence provides a standard benchmark for evaluating our approach. Additionally, it can also ensure that the experimental results that are not affected by other factors. The main idea of k-fold cross-validation is to randomly split the original dataset into k independent subsets. These k subsets share the same information with all categories and contain the same number of instances. The entire procedure of k-fold cross-validation requires k independent algorithm runs, and in each k-fold cross-validation run, one of the k subsets is selected as a test set for classifier evaluation, and the remaining k-1 independent subsets are employed as a training set to model a classifier. During all k runs of k-fold cross-validation, each subset has a chance to be selected as the validation set, with the remaining k-1 subsets being employed as the training set. After all k-fold cross-validation runs, the achieved results are averaged. In the handwritten recognition experiment, 5-fold cross-validation is adopted to randomly split the MNIST dataset, which originally consists of 6000 training samples and 10000 testing samples, into 5 independent subsets such that each subset is comprised of 14000 samples and shares information with ten categories. In each 5-fold cross-validation run, one of the 5 independent subsets is selected as the test set, and the remaining 4 independent subsets are employed as the training set. After all 5-fold cross-validation runs, we can obtain the average and standard deviation of the achieved results from the five instances of 5-fold cross-validation.

Table 2 shows the classification accuracy of candidate classifiers generated by solutions (network configurations) from the proposed approach using the PSO algorithm and random manner for five runs of 5-fold cross-validation on the MNIST dataset. As shown in the achieved results in rows 1–20 of the table, we can observe that the use of different candidate solutions (network configurations) for training of the deep learning architectures usually derives a different classification model with different generalization performance. In addition, to compare the performance of the DNN classifiers generated by solutions of DNN-PSO and DNN-NONPSO, the solutions generated by DNN-PSO, which are converted into network configurations to train DNN classifiers, are superiority to the solutions generated by DNN-NONPSO. This indicates that the PSO algorithm in combination with the steepest gradient descent algorithms, which results in the combination of their two advantages of global and local global exploration abilities, can usually determine a more appropriate network structure and hyperparameters for the individual DNN classifier. These determined optimal network configurations usually perform better in DNN network training, and the DNN classifiers trained by these hyperparameters would provide better generalization ability than the random method. The last two lines display the average and standard deviation of the classification accuracies and support the above conclusion.

**Table 2:** The prediction results of candidate classifiers generated from the final PSO (*pbest*) solutions and randomly generated solutions on the testing dataset using neural network classifiers with two processing layers for the 5-fold cross-validation on the MNIST dataset. (Here, Avg denotes the average prediction result across all candidate classifiers, and N of c denotes the number of combined classifiers).

	DNN-PSO					DNN-NONPSO				
N of c	Fold-1	Fold-2	Fold-3	Fold-5	Fod-5	Fold-1	Fold-2	Fold-3	Fold-4	Fold-4
1	0.9785	0.9801	0.9770	0.9746	0.9808	0.9774	0.9708	0.9738	0.9750	0.1121
2	0.9698	0.9804	0.9788	0.9705	0.9775	0.9714	0.9674	0.9779	0.9733	0.9754
3	0.9774	0.9791	0.9807	0.9726	0.9791	0.9797	0.9348	0.9764	0.9764	0.9786
4	0.9816	0.9794	0.9764	0.9779	0.9831	0.9755	0.9787	0.9769	0.9741	0.9798
5	0.9795	0.9797	0.9781	0.9764	0.9779	0.9809	0.9768	0.9790	0.9720	0.9761
6	0.9791	0.9802	0.9736	0.9775	0.9784	0.9794	0.9754	0.9776	0.9745	0.9768
7	0.9799	0.9761	0.9784	0.9728	0.9786	0.9782	0.9766	0.9768	0.9663	0.9823
8	0.9807	0.9762	0.9692	0.9692	0.9804	0.9759	0.9771	0.9761	0.9551	0.9776
9	0.9795	0.9776	0.9756	0.9736	0.9809	0.9816	0.9769	0.9731	0.9652	0.9794
10	0.9759	0.9804	0.9776	0.9781	0.9785	0.9776	0.9792	0.9768	0.9741	0.9771
11	0.9805	0.9801	0.9779	0.9740	0.9792	0.9802	0.9761	0.9735	0.9735	0.9814
12	0.9751	0.9801	0.9756	0.9732	0.9763	0.9767	0.9816	0.9754	0.9755	0.9784
13	0.9817	0.9789	0.9761	0.9758	0.9739	0.9784	0.9774	0.9773	0.9741	0.9790
14	0.9745	0.9781	0.9781	0.9759	0.9809	0.9711	0.9735	0.9719	0.9721	0.9787
15	0.9797	0.9798	0.9776	0.9761	0.9826	0.9784	0.9797	0.9660	0.9755	0.9783
16	0.9789	0.9782	0.9769	0.9748	0.9821	0.9747	0.9797	0.9651	0.9731	0.9797
17	0.9761	0.9801	0.9786	0.9741	0.9815	0.9744	0.9531	0.9781	0.9749	0.9376
18	0.9751	0.9772	0.9787	0.9729	0.9789	0.9795	0.9739	0.9779	0.9685	0.9802
19	0.9792	0.9794	0.9774	0.9767	0.9807	0.9774	0.9766	0.9749	0.9749	0.9577
20	0.9765	0.9782	0.9763	0.9726	0.9749	0.9791	0.9664	0.9601	0.9734	0.9784
Avg	0.9780	0.9790	0.9769	0.9745	0.9793	0.9774	0.9726	0.9742	0.9721	0.9322
Std	0.0029	0.0013	0.0023	0.0023	0.0024	0.0029	0.0110	0.0050	0.0050	0.1933

Table 3 displays the prediction results of the final classification models (the ensemble model and the individual DNN classifier) constructed by the proposed approach respectively using the PSO algorithm and random method for five runs of 5-fold cross-validation on the MNIST dataset. As shown in the table, an ensemble model and individual DNN classifiers constructed by the solutions (*pbest*) and solution (*gbest*) of the PSO algorithm have achieved the best classification accuracies of 0.9849 and 0.9795, respectively. The above achieved results show that the ensemble model can provide better generalization capability than the individual DNN classifier. In addition, although the individual DNN classifier is constructed by the best solution (*gbest*) of the PSO algorithm, the classification accuracy achieved by the ensemble model combining 20 individual DNN classifiers is still superior to the best individual classifier. The main reason may be because combining evidence across multiple DNN classifiers of the ensemble model may result in the construction of a classification model in which some poorly performing DNN classifiers would be ignored by most of the well-performing DNN classifiers during the prediction phase. The more specific case can be seen in the ensemble without using the PSO-based optimization scheme. Table 3 also shows the average and standard deviation of the classification accuracy of the ensemble model and the individual classifier, and the results clearly confirm the superiority of the ensemble model compared to the individual DNN classifier. In addition, the computational times required to train an ensemble model and an individual DNN classifier using the PSO-based approach and random method are displayed in the last columns of Table 3. It can be seen that training of an ensemble model needs to almost speed computational resources 20 times than the individual DNN classifier. More DNN classifiers are trained to form an ensemble model that would require more computational resources. Moreover, it can also be seen that training an ensemble model and an individual DNN classifier using a PSO-based approach requires less computational time for training the final models than the DNN-NONPSO approach. This may be because a more appropriate network structure with a better hyperparameter configuration not only derives a well-performed DNN classifier with better generalization capability but also accelerates the convergence of the training phase.

**Table 3:** The prediction results of the final classification models (ensemble model and individual DNN classifier) of the proposed approach using the PSO algorithm and random method for the 5-fold cross-validation runs on the MNIST Dataset. (Here, Avg and Std denote the average and standard deviation of the prediction results, and Ensemble and Individual denote the prediction results achieved by the ensemble model and the individual DNN classifier, respectively. Time 1 and Time 2 denote the computational times of the training phases of the ensemble model and the individual DNN classifier, respectively.)

	The proposed approach using PSO				The proposed approach without PSO			
Folds	Ensemble	Individual	Time1	Time2	Ensemble	Individual	Time1	Time2
Fold-1	0.9861	0.9821	1016.4460	54.0736	0.9848	0.9787	940.4149	36.31399
Fold-2	0.9855	0.9781	856.5165	38.7264	0.9840	0.9777	1124.966	51.72183
Fold-3	0.9842	0.9793	892.0217	41.3456	0.9826	0.9774	945.8587	48.6305
Fold-4	0.9820	0.9775	859.5301	34.9860	0.9796	0.9737	892.9707	37.32175
Fold-5	0.9870	0.9805	937.8662	58.9434	0.9852	0.9796	1070.725	31.24267
Avg	0.9850	0.9795	912.4761	45.6150	0.9832	0.9774	994.9871	41.04615
Std	0.0019	0.0019	59.6783	9.2504	0.0020	0.0020	87.66965	7.795707

Table 4 presents the detailed experimental results of the ensemble models when combining different numbers of DNN classifiers for five runs of 5-fold cross-validation on the MNIST dataset. The table presents results achieved by the ensemble models, which are constructed by directly choosing a certain number of DNN classifiers from the 20 independent DNN classifiers that have been initialized and trained by the solutions representing network configurations generated by using DNN-PSO and DNN-NONPSO approaches, respectively, without training new neural networks. The achieved results indicate that combining evidence from *n* trained DNN classifiers may derive a different ensemble model with different generalization capabilities when varying *n* from 2 to *n*. In addition, the prediction accuracy of the ensemble model is increased when the number of members (trained DNN classifiers) is increasing. The above results show that, the more DNN classifiers that are selected from the trained classifiers, employed to construct an ensemble model may provide better generalization capability than a few DNN classifiers combined. However, the table presented results that also show that the prediction accuracy of the ensemble model with a certain number of DNN classifiers has almost achieved the highest accuracy or has provided only a slight improvement when the number of DNN classifiers is increased. The computational times for training and testing of an ensemble model are considerable when choosing more DNN classifiers.

**Table 4:** The prediction results when using ensemble models with different numbers of classifiers.

	Ensemble model using PSO					Ensemble model without PSO				
N of c	Fold-1	Fold-2	Fold-3	Fold-5	Fold-5	Fold-1	Fold-2	Fold-3	Fold-4	Fold-5
2	0.9749	0.9791	0.9765	0.9764	0.9759	0.9770	0.9690	0.9776	0.9709	0.3046
4	0.9821	0.9831	0.9804	0.9796	0.9820	0.9814	0.9775	0.9796	0.9777	0.9801
6	0.9832	0.9844	0.9821	0.9808	0.9844	0.9834	0.9798	0.9793	0.9794	0.9838
8	0.9851	0.9847	0.9834	0.9814	0.9849	0.9836	0.9818	0.9805	0.9803	0.9844
10	0.9859	0.9851	0.9837	0.9813	0.9862	0.9843	0.9832	0.9814	0.9802	0.9850
12	0.9856	0.9854	0.9836	0.9816	0.9869	0.9851	0.9837	0.9817	0.9798	0.9853
14	0.9861	0.9853	0.9836	0.9813	0.9868	0.9856	0.9842	0.9821	0.9796	0.9857
16	0.9861	0.9854	0.9838	0.9819	0.9871	0.9849	0.9843	0.9826	0.9794	0.9857
18	0.9864	0.9856	0.9841	0.9814	0.9874	0.9846	0.9843	0.9828	0.9796	0.9856
Avg	0.9839	0.9842	0.9824	0.9806	0.9840	0.9833	0.9809	0.9808	0.9785	0.9089
Std	0.0037	0.0021	0.0025	0.0017	0.0037	0.0027	0.0050	0.0017	0.0030	0.2266

**Fig 4:**
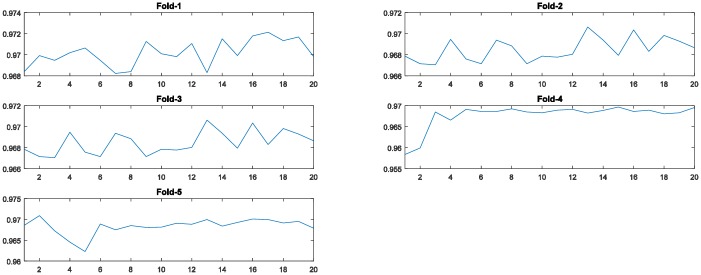
The training accuracy (fitness value) of the PSO algorithm at each generation across all generations for the 5-fold cross-validation runs on the MNIST dataset. (The horizontal axis and the vertical axis represent the number of generations of the PSO algorithm and training accuracy, respectively.)

Fig 4 displays the fitness values generated by the PSO algorithm during the evolutionary procedure. As shown in these graphs, in all five-fold runs, the fitness curves increase with increasing generations until a certain number, demonstrating that the PSO algorithm is able to find better solutions by its evolutionary procedure.

**Fig 5:**
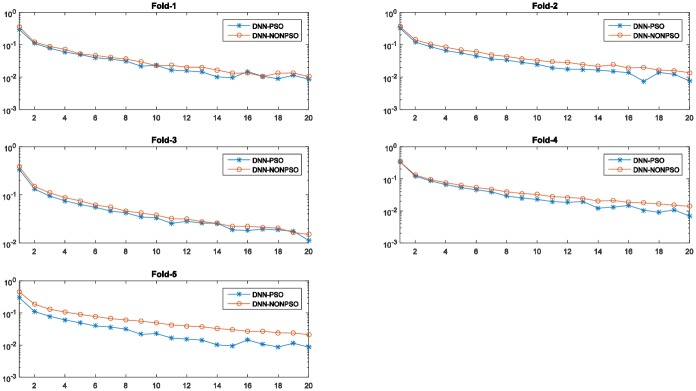
The training error values (loss values) of the network training with 20 epochs of the individual classifier using the PSO algorithm and random method for five runs of 5-fold cross-validation on the MNIST dataset.

Fig 5 displays the training errors (training loss values) generated by the individual DNN classifier initialized by the solution of the PSO-based optimization scheme and random method, respectively. As shown in these graphs, all training loss values generated by five runs of 5-fold cross-validation indicate that the individual classifier initialized by the best solution (*gbest*) of the PSO algorithm and then trained with the solution (*gbest)* corresponding to the hyperparameter configuration can produce a decreasing curve during the 20 epochs. In addition, the training loss values generated by the individual classifier initialized and trained by the solution (*gbest*) of the PSO algorithm not only achieved the lowest loss value at each epoch compared with the random solution but also obtained the lowest loss value on the latest training phase. The observed results also indicate that a more appropriate network structure with a better hyperparameter configuration can improve the performance in the network training phase and of the final achieved model, and the PSO algorithm in combination with the steepest gradient descent algorithm can utilize their global and local exploration capabilities to automatically discover the optimal network configuration without any prior knowledge.

### 4.3 Results and analysis on the KCD dataset

In this subsection, we evaluate the prediction performance of the proposed approach in solving the regression problem. To solve the regression problem, the prediction of biological activity is an important issue in computational biology fields and helps researchers to improve their work on drug discovery. In this experiment, KCD datasets are suitable for evaluating performance of various algorithms and investigating the effectiveness of the proposed approaches on predicting biological activity. Before performing any training procedures on KCD datasets, we need to preprocess the data. The details of the above process are illustrated as follows. The high-dimensional descriptors (features) that exist in each sample are difficult for training of a deep learning architecture, and hence this study uses the Principal Component Analysis (PCA) technique to reduce the number of descriptors to an appropriate amount so that the deep learning architecture can efficiently process them. All 15 targets of the KDD datasets were processed with PCA for feature reduction. After the above process, the remaining descriptors of 15 targets of the KCD datasets are scaled into the range of (0, 1). For this regression experiment, all parameter domains were set as follows. The number of processing layers was two, and each processing layer’s neurons were initialized by a randomly generated number in the range of (100, 150). The sigmoid function was employed to compute the activation value of the neuron. The number of epochs for population evaluation (performing small epochs of mini-batch learning to evaluate classifiers generated by the population of the PSO algorithm) was set to 30 and for final network training was set to 100. The batch size was set to 100. The domains of the learning rate, momentum, decay, dropout rate were set to the ranges (0.01, 0.1), (0.1, 0.9), (0.00001, 0.0001), and (0.1, 0.9), respectively. For evaluating the performance of the proposed approach on each biological dataset, a distribution ratio is used to randomly divide all of the samples of each dataset into two subsets, where 80% of the samples were employed to train a final network and the remaining samples were employed as a testing set for evaluation.

The performance of the 20 independent DNN classifiers generated by the solutions (*pbest*) of the PSO algorithm and random solutions for 15 datasets is presented in Tables 5, 6 and 7, providing the detailed experimental results for datasets 1–5, 6–10, and 11–15, respectively. It can be observed that DNN classifiers whose network structures with hyperparameters are determined using the PSO algorithm and trained using the steepest gradient descent algorithm yield better performance than the random approach. In addition, as shown in these tables, the DNN classifiers that were initialized and trained by the solutions generated by the PSO algorithm almost achieved the best prediction results in terms of the MAPE for each biological activation dataset. The last two rows of these tables show not only that the proposed approach using the PSO algorithm gives the best DNN classifiers with good classification performance but also that these DNN classifiers yielded the lowest standard deviation. Based on the above results, it is also shown that the use of PSO in combination with steepest gradient descent algorithms can determine more appropriate network configurations (network structure and hyperparameter configuration) for initializing and training the final model with good generalization performance to solve regression problems.

**Table 5:** The prediction results of candidate DNN classifiers generated by the solutions (*pbest*) and solutions of the DNN-PSO and DNN-NONPSO approaches, respectively, for one run of the 1–5 biological activity datasets.

	DNN-PSO					DNN-NONPSO				
N of c	3A4	CB1	DPP4	HIVINT	HIVPROT	3A4	CB1	DPP4	HIVINT	HIVPROT
1	0.2005	1.2965	1.5144	0.2405	3.4386	0.3381	1.3691	1.4583	0.2414	3.9081
2	0.3376	1.4552	1.4763	0.1172	1.4011	0.3399	1.4359	1.5188	0.2223	3.8855
3	0.2206	1.5383	1.4856	0.1674	3.9444	0.3371	1.4346	1.6042	0.1756	3.5860
4	0.3395	1.3436	1.5052	0.2972	3.4178	0.3348	0.6966	1.4594	0.1975	3.7107
5	0.2049	1.7389	1.4795	0.1119	3.5055	0.2191	1.3310	1.4972	0.1910	3.5011
6	0.3382	1.3303	1.4629	0.1120	3.4979	0.3464	1.3935	1.7628	0.1625	4.3691
7	0.1970	1.3033	1.4558	0.1191	3.4594	0.3348	1.4196	1.4600	0.1252	3.3564
8	0.2094	1.4704	1.4585	0.1123	3.4970	0.3383	1.3324	2.3290	0.1923	3.3019
9	0.3360	1.3613	1.6974	0.1137	3.5384	0.3419	1.5481	1.4730	0.1786	3.7017
10	0.3404	1.3693	2.1176	0.1153	4.0036	0.3424	1.3394	1.5663	0.1658	3.4862
11	0.2335	1.3537	1.6197	0.2206	3.3211	0.3349	1.6895	2.3901	0.1220	3.4698
12	0.3359	1.4235	1.7801	0.2091	3.5891	0.3369	1.3740	1.4571	0.2185	1.2337
13	0.3348	1.3573	1.5798	0.1160	3.8871	0.3357	1.3112	1.4908	0.2015	4.5231
14	0.1983	1.3684	1.4993	0.2281	3.3522	0.3415	1.3111	1.4570	0.1643	3.9276
15	0.2201	1.7648	1.9797	0.1112	3.5857	0.3543	1.3444	1.6175	0.2001	3.4792
16	0.3348	1.3097	1.4814	0.2679	3.4605	0.3358	1.4377	1.4901	0.1152	3.7925
17	0.1984	1.2908	1.4541	0.2122	3.7491	0.3499	1.2944	1.5022	0.1787	3.4836
18	0.3365	1.3842	1.4541	0.1173	3.8058	0.3417	1.4933	1.5069	0.2047	3.4937
19	0.1989	1.5187	1.4591	0.1230	3.6477	0.3377	1.3387	1.4607	0.1745	3.4273
20	0.2194	1.5273	1.4542	0.2543	3.6890	0.3487	1.5820	1.4989	0.2167	3.5062
Avg	0.2667	1.4253	1.5707	0.1683	3.4895	0.3345	1.3738	1.6000	0.1824	3.5572
Std	0.0659	0.1355	0.1870	0.0649	0.5282	0.0277	0.1894	0.2704	0.0338	0.6331

**Table 6:** The prediction results of candidate DNN classifiers generated by the solutions (*pbest*) and solutions of the DNN-PSO and DNN-NONPSO approaches, respectively, for one run of the 6–10 biological activity datasets.

	DNN-PSO					DNN-NONPSO				
N of c	LOGD	METAB	NK1	OX1	OX2	LOGD	METAB	NK1	OX1	OX2
1	1.2482	1.2736	1.6554	1.0266	1.0777	1.2497	3.8073	0.9765	1.1638	1.9109
2	1.2408	1.2771	1.3910	1.0542	1.7795	1.2632	3.4689	0.4729	1.0406	1.9322
3	1.2406	1.2518	1.8322	1.0686	1.7323	1.2810	3.7857	1.7560	1.0377	1.8696
4	1.2675	0.4726	1.3428	1.0271	1.6467	1.2491	3.8208	0.9928	1.1308	1.0476
5	1.2496	0.4648	1.4885	1.5774	1.6804	1.2649	3.7294	0.5834	1.0956	1.0860
6	1.3532	1.2502	1.1518	1.0415	2.0970	1.2406	4.4178	1.4813	1.1960	1.1095
7	1.2599	0.4203	1.2815	1.2564	2.0083	1.2411	3.5244	2.1845	1.0281	1.6198
8	1.2498	0.4264	0.8911	1.1450	1.7527	1.2991	3.4270	1.4342	1.1475	2.0238
9	1.2447	1.2408	1.3539	1.1338	2.1255	1.3345	3.5422	1.6333	1.0672	2.0282
10	1.2424	0.7727	1.6205	1.0674	1.7241	1.2406	3.4181	0.5948	1.0979	1.9676
11	1.2470	0.4374	2.6143	1.0499	1.6180	1.2406	3.7073	1.5808	1.2062	1.7595
12	1.2463	1.2726	1.5508	1.2470	1.9858	1.3086	3.3049	2.1852	1.0790	1.0359
13	1.2604	1.2421	1.6819	1.2966	1.7688	1.2433	4.0172	1.6795	1.0230	1.8745
14	1.2424	1.2422	1.8027	1.0646	1.6546	0.4445	3.4941	0.7625	1.0265	1.8699
15	1.2441	1.2919	1.4408	1.0655	2.0290	1.2555	3.6298	1.1430	0.5670	1.8007
16	1.2475	1.2968	1.3721	1.0256	1.8031	1.3056	3.7405	1.7333	1.0231	1.8184
17	1.2788	1.2584	1.5148	1.0316	2.0049	1.2460	3.8127	1.4720	1.2042	1.5613
18	1.2440	1.2425	1.4503	1.0870	1.7276	1.2429	3.9439	1.5463	1.0480	1.8485
19	1.2465	0.4372	1.7415	0.5934	1.8387	1.2741	3.4115	0.5149	1.0456	1.8577
20	1.2448	1.2542	1.2924	1.0756	1.6917	1.2778	4.2795	1.6047	1.0486	1.8503
Avg	1.2549	0.9913	1.5235	1.0967	1.7873	1.2251	3.7142	1.3166	1.0638	1.6936
Std	0.0251	0.3841	0.3425	0.1787	0.2305	0.1858	0.2907	0.5305	0.1330	0.3391

**Table 7:** The prediction results of candidate DNN classifiers generated by the solutions (*pbest*) and solutions of the DNN-PSO and DNN-NONPSO approaches, respectively, for one run of the 11–15 biological activity datasets.

	DNN-PSO					DNN-NONPSO				
N of c	PGP	PPB	RAT_F	TDI	THROMBIN	PGP	PPB	RAT_F	TDI	THROMBIN
1	0.2738	0.6853	0.2775	0.1747	4.8488	1.9109	0.3755	0.6063	0.2070	4.4219
2	0.2026	0.6042	0.2617	0.2301	4.5214	1.9322	0.2415	0.6051	0.2007	4.9896
3	0.2548	0.6371	0.2490	0.2383	4.4301	1.8696	0.5443	0.8732	0.2080	5.1188
4	0.3912	0.6024	0.2765	0.2142	5.3115	1.0476	0.3589	0.2847	0.2006	4.4378
5	0.4672	0.6152	0.2611	0.2064	4.4291	1.0860	0.3687	0.6669	0.2038	4.8855
6	0.3548	0.7352	0.3050	0.2138	4.4711	1.1095	0.3824	0.6020	0.2038	4.4236
7	0.3662	0.6631	0.2636	0.2021	4.4412	1.6198	0.3546	0.6051	0.2193	4.4287
8	0.2736	0.7228	0.2732	0.1745	4.4608	2.0238	0.3676	0.6366	0.2133	4.5431
9	0.3885	0.6282	0.2411	0.2003	4.4354	2.0282	0.3931	0.6122	0.2045	4.7623
10	0.2923	0.6109	0.3150	0.1782	4.4451	1.9676	0.3778	0.6919	0.2032	4.4858
11	0.2338	0.9068	0.4149	0.2031	4.5070	1.7595	0.3622	0.7988	0.2012	4.7073
12	0.3790	0.6176	0.2659	0.2067	4.4281	1.0359	0.4280	0.7561	0.2013	4.6890
13	0.2250	0.7066	0.2566	0.1786	4.4405	1.8745	0.2856	0.6019	0.2774	4.4220
14	0.2303	0.6918	0.2814	0.2016	4.5464	1.8699	0.3579	0.7507	0.2004	4.4267
15	0.2309	0.7135	0.2739	0.2313	4.4768	1.8007	0.3809	0.8010	0.2135	4.5384
16	0.3664	0.6614	0.2837	0.1537	5.1392	1.8184	0.3588	0.6276	0.2097	4.4223
17	0.3545	0.7798	0.2711	0.2041	4.4594	1.5613	0.3576	0.7625	0.2038	4.4722
18	0.3587	0.8724	0.2446	0.1763	4.8130	1.8485	0.3548	0.6245	0.2551	1.7893
19	0.1974	0.6896	0.2973	0.2006	4.4302	1.8577	0.3575	0.8291	0.2297	4.8561
20	0.2872	0.6082	0.2640	0.2426	4.4240	1.8503	0.3548	0.6249	0.2032	4.4759
Avg	0.3064	0.6876	0.2789	0.2016	4.5730	1.6936	0.3681	0.6680	0.2130	4.4648
Std	0.0764	0.0856	0.0371	0.0236	0.2542	0.3391	0.0561	0.1265	0.0200	0.6654

Table 8 shows the prediction results of the ensemble model with different numbers of s using DNN-PSO and DNN-NONPSO approaches for 15 KCD datasets. As shown in the table, in most cases, combining more DNN classifiers may provide better prediction performance. In addition, selecting the 18 DNN classifiers as the members of an ensemble model may result in a prediction phase in which the outcomes generated by the ensemble model are only slight improvements compared with the rest of the models. Table 9 shows the prediction results of the ensemble model and the individual DNN classifier generated by the solutions (*pbest*) and (*gbest*) of the PSO algorithm and random approach, respectively, for 15 KCD datasets. The last two rows of the table present the average and standard deviation of accuracies generated by the two different approaches on 15 KCD datasets, respectively. We can observe that the ensemble model and the individual DN classifiers constructed by the PSO-based optimization scheme not only achieved better average prediction results but also yielded the lowest standard deviation of accuracy. In addition, using the solutions of the PSO algorithm to initialize the ensemble model and individual DNN classifier and then train these neural networks with their corresponding hyperparameter configurations requires less computational time to implement the final models compared to the random approach. Based on the above results, it is shown that the optimal network configurations can simultaneously improve the classification performance and training efficiency compared with randomly selected configurations.

**Table 8:** The classification accuracy of the ensemble models with different number of DNN classifiers generated using the PSO-based optimization scheme and random approach, respectively, for 15 targets of the KCD datasets.

	Ensemble model using PSO					Ensemble model without PSO				
N of c	3A4	CB1	DPP4	HIVINT	HIVPROT	3A4	CB1	DPP4	HIVINT	HIVPROT
2	0.2408	1.3271	1.4697	0.2821	3.5933	0.3349	1.4240	1.4757	0.2217	3.6719
4	0.2805	1.3082	1.4546	0.2238	3.5899	0.3368	1.3600	1.4850	0.1641	3.6451
6	0.2977	1.3529	1.4744	0.2312	3.5361	0.3393	1.3473	1.4558	0.1677	3.6530
8	0.3065	1.3609	1.4720	0.2283	3.5781	0.3375	1.3447	1.4541	0.1770	3.2536
10	0.3118	1.3609	1.4544	0.2037	3.5703	0.3358	1.3642	1.4711	0.1596	3.2910
12	0.2827	1.3682	1.4547	0.1748	3.5611	0.3362	1.3720	1.4545	0.1634	3.2988
14	0.2683	1.3587	1.4542	0.1597	3.5483	0.3364	1.3766	1.4550	0.1531	3.3537
16	0.2563	1.3716	1.4544	0.1475	3.5359	0.3240	1.2832	1.4553	0.1574	3.3833
18	0.2451	1.3825	1.4544	0.1375	3.3601	0.3250	1.2985	1.4546	0.1610	3.4192
D 6–10	LOGD	METAB	NK1	OX1	OX2	LOGD	METAB	NK1	OX1	OX2
2	1.2459	1.2533	1.5495	1.0476	1.1977	1.2412	3.8735	1.2172	1.0941	1.8790
4	1.2417	1.2628	1.4946	1.0361	1.4692	1.2494	3.8236	1.3932	1.0784	1.7436
6	1.2409	1.2613	1.5328	1.0440	1.5704	0.9966	3.7244	1.1725	0.9277	1.7718
8	1.2408	1.2563	1.5530	1.0774	1.6382	1.0579	3.6622	1.3327	0.9531	1.5955
10	1.2406	1.2554	1.6461	1.0732	1.6395	1.0913	3.6360	1.2294	0.9849	1.6432
12	1.2409	1.2565	1.5326	1.0823	1.6807	1.1138	3.6066	1.2755	1.0030	1.6983
14	1.2431	1.1444	1.4820	1.0870	1.7258	1.1306	3.6410	1.3427	1.0139	1.5653
16	1.2442	0.9951	1.4733	1.0970	1.7146	1.1440	3.6564	1.2314	1.0253	1.4438
18	1.2435	0.8677	1.4865	1.0924	1.7188	1.1561	3.6507	1.1718	1.0201	1.4862
D 6–10	PGP	PPB	RAT_F	TDI	THROMBIN	PGP	PPB	RAT_F	TDI	THROMBIN
2	0.3594	0.6768	0.2622	0.2096	4.8307	0.3611	0.6033	0.2800	0.2252	2.2657
4	0.3547	0.6552	0.2731	0.2012	4.4463	0.3564	0.6159	0.2828	0.2142	3.1515
6	0.3613	0.6500	0.2739	0.1817	4.4373	0.3546	0.6494	0.2591	0.2097	3.5472
8	0.3632	0.6600	0.2722	0.1862	4.4362	0.3308	0.6480	0.2642	0.2021	3.7272
10	0.3437	0.6656	0.2691	0.1888	4.4416	0.3212	0.6625	0.2641	0.2010	3.8545
12	0.3183	0.6345	0.2751	0.1811	4.4340	0.3300	0.6544	0.2646	0.2017	3.9463
14	0.2995	0.6475	0.2671	0.1757	4.4297	0.3340	0.6393	0.2660	0.2017	4.0111
16	0.2880	0.6425	0.2614	0.1723	4.4419	0.3374	0.5984	0.2577	0.2016	4.0668
18	0.2768	0.6471	0.2554	0.1734	4.4440	0.3255	0.6069	0.2594	0.2017	4.1051

**Table 9:** The prediction results of the ensemble model and the individual DNN classifier using the PSO-based optimization scheme and random approach for 15 targets of the KCD datasets. (Result1 and Result2 denote the classification accuracy of the ensemble model and the individual classifier, respectively. In addition, Time1 and Time2 are computational times of the network training phase for the ensemble model and the individual DNN classifier, respectively.).

	DNN-PSO				DNN-NONPSO			
DataSet	Result1	Result2	Time1	Time2	Result1	Result2	Time1	Time2
1	0.2374	0.2113	821.6200	25.4780	0.3264	0.3354	896.1800	53.0870
2	1.3937	1.4728	1127.9000	44.9750	1.3110	1.4044	818.1400	21.1910
3	1.4544	1.5821	910.8900	35.3510	1.4548	1.5043	588.0000	26.4020
4	0.1304	0.1108	101.5000	4.1661	0.1638	0.1815	107.2100	5.7587
5	3.3894	3.7967	191.3800	10.9170	3.4216	3.5751	178.8900	8.7294
6	1.2430	1.2409	1441.0000	39.8040	1.1663	1.2559	1011.3000	45.5930
7	0.7825	0.5313	725.5000	26.9390	3.6589	3.3978	227.8900	10.1810
8	1.4879	2.1590	1024.4000	59.3520	1.1070	1.1072	582.8500	45.6880
9	1.0471	1.0256	559.6900	30.7540	1.0225	1.0971	427.1600	18.5420
10	1.7226	1.7181	1168.5000	64.7090	1.5182	1.7745	536.9000	33.1380
11	0.2660	0.3565	355.0300	13.9510	0.3265	0.3869	291.2000	22.1710
12	0.6457	0.7246	686.6400	33.0760	0.6088	0.6248	458.0700	23.7750
13	0.2509	0.2742	405.6200	14.1600	0.2567	0.2751	397.5300	19.9690
14	0.1669	0.1464	244.5200	10.0500	0.1973	0.2006	273.6000	15.9590
15	4.4391	4.4346	459.4700	21.2980	4.1438	4.6492	366.1600	20.5650
Avg	1.2438	1.3190	681.5700	28.9990	1.3789	1.4513	477.4000	24.7170
Std	1.2286	1.3079	398.1600	17.8360	1.3156	1.3768	265.8700	14.0280

**Fig 6:**
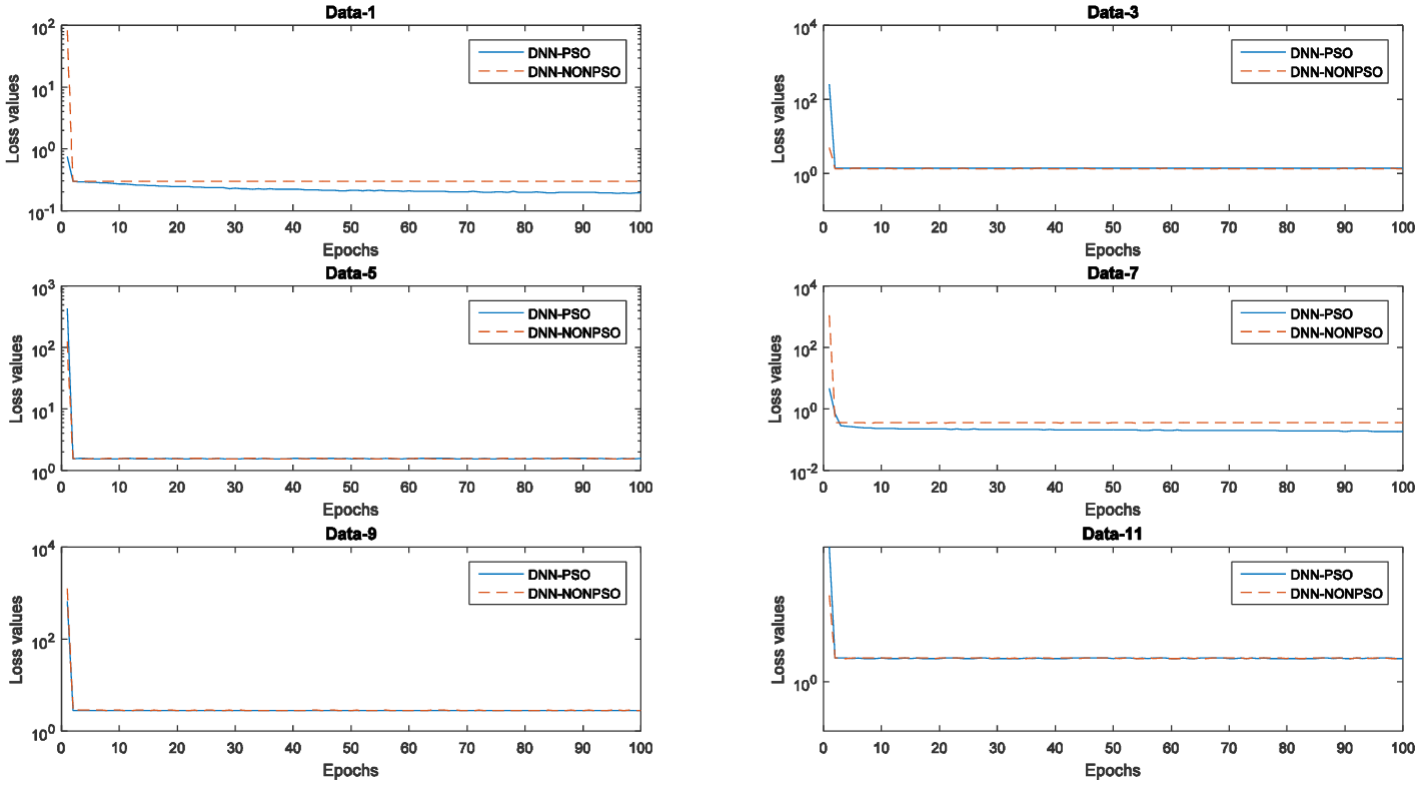
The training error values (loss values) of the network training using three hidden layers with 100 epochs of the individual classifier using the PSO algorithm and random method for 1–6 KCD datasets.

**Fig 7:**
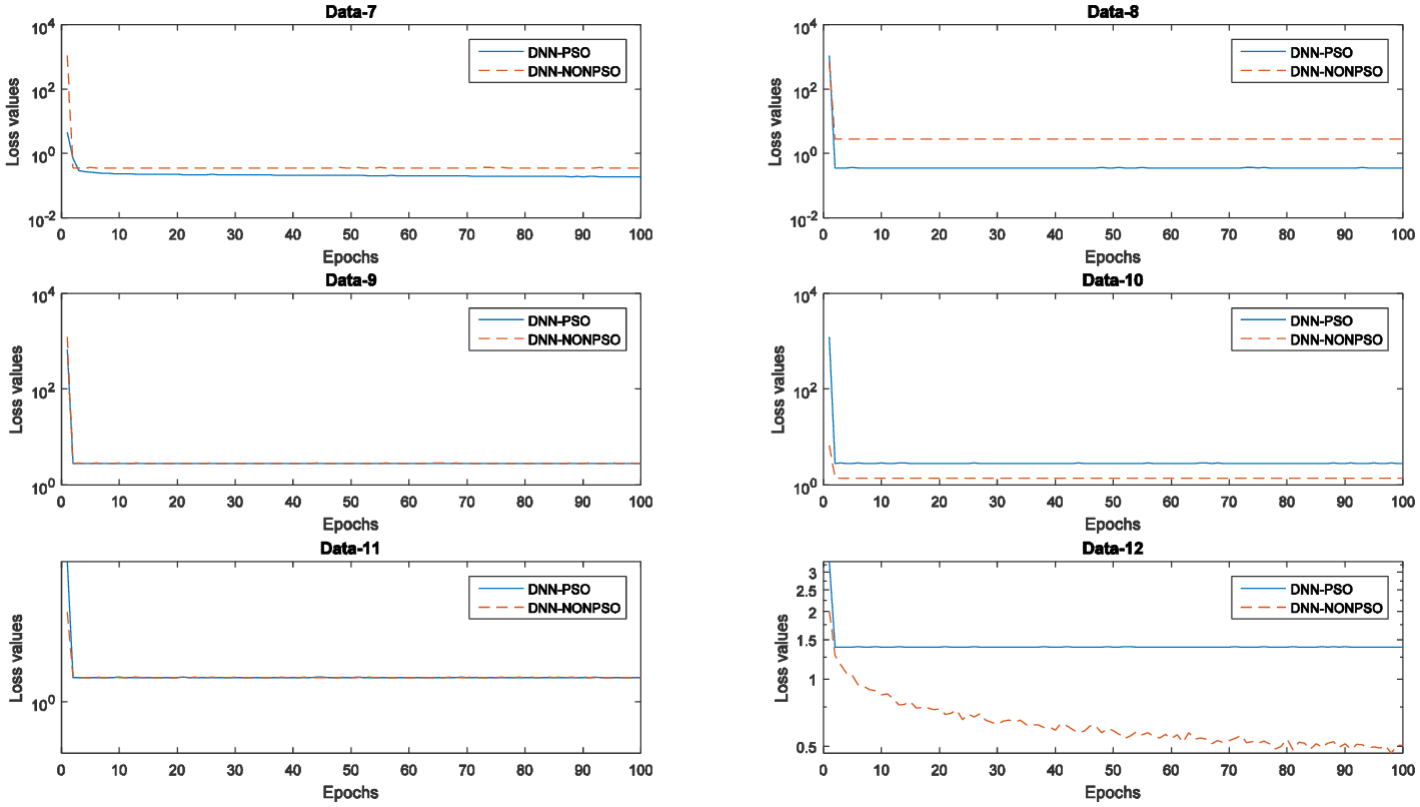
The training error values (loss values) of the network training using three hidden layers with 100 epochs of the individual classifier using the PSO algorithm and random method for 7–12 KCD datasets.

**Fig 8:**
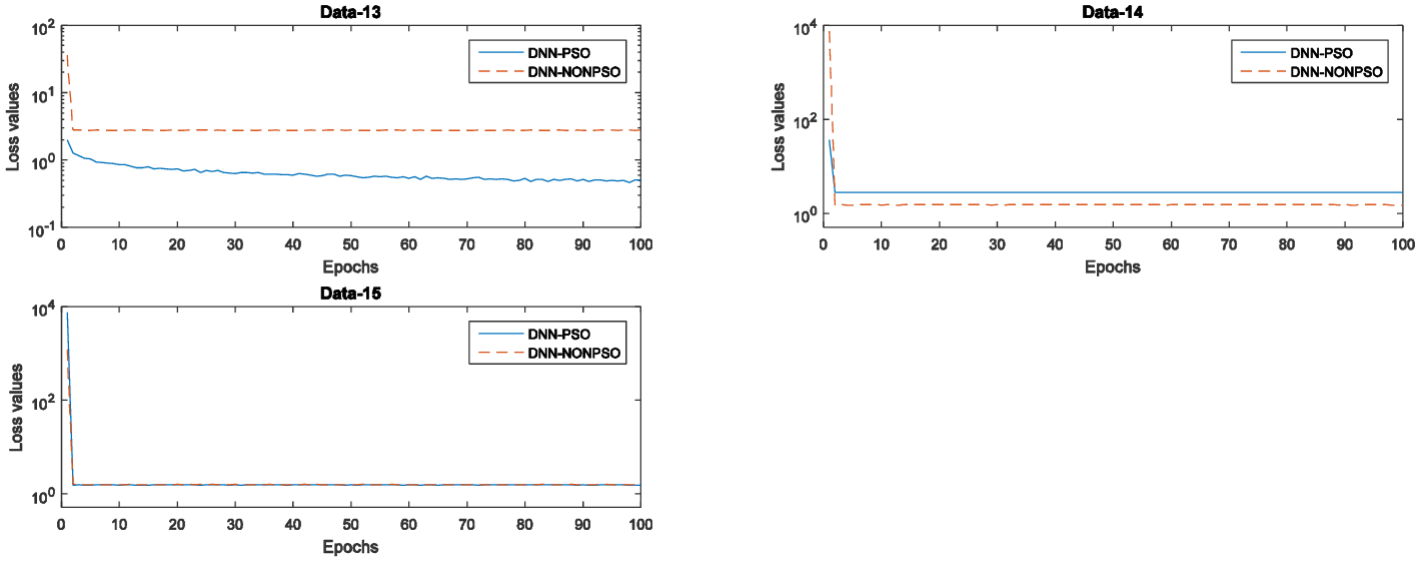
The training error values (loss values) of the network training using three hidden layers with 100 epochs of the individual classifier using the PSO algorithm and random method for 13–15 KCD datasets.

Figs 6, 7 and 8 respectively display the training errors of the individual classifier generated by the solution (*gbest*) of the PSO algorithm and random configuration for 15 KCD datasets. As shown in these graphs, *gbest* is employed to initialize a DNN classifier and then train the classifier with its corresponding hyperparameter configuration, which generates lower training curves than the random configuration. In addition, not only did the individual classifier using the DNN-PSO approach generate the lowest training curves, but it also obtained the lowest final training error in the last training phase.

### 4.4 The investigation of deeper networks using the proposed approach

In this subsection, we investigate the influence of the use of the PSO-based optimization scheme for deeper neural networks and evaluate the effectiveness of these trained DNN classifiers on the MNIST dataset. For the experimental parameter setting, the search ranges for the optimization parameters, and the parameter configurations for the PSO algorithm and final network training adopt the same settings as in the previous experiment. In addition, we adopted three hidden layers to construct a deep neural network, and the number of hidden neurons of each hidden layer was searched for in the same domain. The five runs of the 5-fold cross-validation procedure with mini-batch learning are performed to train DNN classifiers and evaluate them. After five runs, we can obtain the final results by calculating the average and standard deviation of these achieved results. Table 10 shows the detailed classification results of 20 independent DNN classifiers with three hidden layers generated by the solutions (*pbest*) of the PSO algorithm and random approach, respectively, for five runs of 5-fold cross-validation on the MNIST dataset. The same phenomenon can be seen in this table: use of the optimal network configurations that were expressed by solutions of the PSO algorithm to construct DNN classifiers outperforms the classifiers trained using randomly generated configurations. The last two rows of the table show the average and standard deviation of these results within the table. To compare the classification accuracies generated by DNN classifiers with three and two hidden layers, the DNN classifiers perform better when using two hidden layers. This may be because a deeper-level neural network generally has a huge number of adjustable parameters (weights and biases), requiring more training time or computational resources to allow the model to fit the training data. In. The same training parameters (epochs and batch size) that have been employed in the training network with two hidden layers in the previous experiments are now used to train the network with three hidden layers. As a result, this training phase may be insufficient, and more epochs for network training may allow the deeper neural network to reach or exceed the classification performance of the shallow architecture.

**Table 10:** The prediction results of candidate classifiers generated from the final PSO (*pbest*) solutions and randomly generated solutions on the test dataset using neural network classifiers with two processing layers for the 5-fold cross-validation on the MNIST dataset. (Here, Avg denotes the average prediction result across all candidate classifiers, and N of c denotes the number of combined classifiers.).

	DNN-PSO					DNN-NONPSO				
N of c	Fold-1	Fold-2	Fold-3	Fold-5	Fod-5	Fold-1	Fold-2	Fold-3	Fold-4	Fold-4
1	0.9786	0.9798	0.9697	0.9702	0.9808	0.9496	0.9593	0.9754	0.9677	0.9794
2	0.9767	0.9779	0.9784	0.9716	0.9799	0.9736	0.9750	0.9735	0.9682	0.9766
3	0.9778	0.9767	0.9786	0.9763	0.9791	0.9752	0.9746	0.9749	0.9722	0.3713
4	0.9766	0.9764	0.9746	0.9749	0.9807	0.9666	0.9781	0.9722	0.9716	0.9744
5	0.9758	0.9760	0.9756	0.9728	0.9808	0.9787	0.9741	0.9688	0.9731	0.9789
6	0.9758	0.9760	0.9760	0.9736	0.9736	0.9679	0.2082	0.9731	0.4788	0.2121
7	0.9725	0.9756	0.9749	0.9761	0.9801	0.9793	0.9751	0.9754	0.9643	0.9794
8	0.9745	0.9788	0.9785	0.9764	0.9784	0.9772	0.9296	0.9733	0.9707	0.9683
9	0.9790	0.9797	0.9780	0.9746	0.9759	0.9749	0.9529	0.9732	0.9709	0.9798
10	0.9770	0.9756	0.9760	0.9699	0.9778	0.9786	0.9743	0.9681	0.9661	0.9787
11	0.9776	0.9774	0.9783	0.9753	0.9795	0.9749	0.9755	0.9747	0.9751	0.9734
12	0.9754	0.9775	0.9729	0.9764	0.9786	0.1124	0.9743	0.9680	0.9694	0.9780
13	0.9762	0.9769	0.9726	0.9689	0.9791	0.9743	0.9674	0.9759	0.9674	0.9721
14	0.9756	0.9774	0.9769	0.9767	0.9789	0.9776	0.9741	0.9700	0.9703	0.9802
15	0.9779	0.9771	0.9774	0.9756	0.9814	0.9685	0.9759	0.9695	0.9661	0.9768
16	0.9764	0.9744	0.9782	0.9738	0.9786	0.9725	0.9711	0.9766	0.9697	0.9785
17	0.9789	0.9771	0.9762	0.9742	0.9791	0.9724	0.9783	0.9644	0.9722	0.9747
18	0.9765	0.9718	0.9755	0.9697	0.9781	0.8105	0.9756	0.9770	0.9707	0.9750
19	0.9745	0.9781	0.9780	0.9749	0.9756	0.9741	0.9717	0.9726	0.9701	0.9793
20	0.9781	0.9783	0.9749	0.9717	0.9812	0.9765	0.9736	0.9760	0.9746	0.9792
Avg	0.9766	0.9769	0.9761	0.9737	0.9789	0.9218	0.9319	0.9726	0.9455	0.9083
Std	0.0016	0.0018	0.0024	0.0025	0.0020	0.1941	0.1707	0.0034	0.1099	0.2125

Table 11 shows the prediction results of the ensemble model and the individual DNN classifier generated by the two different approaches for five runs of 5-fold cross-validation on the MNIST dataset. As shown in the table, the solutions achieved by the PSO algorithm employed to construct an ensemble model and individual DNN classifier gave the best classification accuracies of 0.9845 and 0.9777, respectively. These trained ensemble model and individual DNN classifier also yielded the lowest standard deviation for accuracy. For the computation times in the training phase, the DNN-PSO is superiority than DNN-NONPSO. Based on the above results, we can conclude that the proposed approach using the PSO algorithm in combination with the steepest gradient descent algorithm can also determine the optimal solutions for the deeper neural network that achieved the significant results. In addition, to compare the results achieved by DNN classifiers with two and three hidden layers, the difference between them is smaller. This may be because the optimal network configuration can overcome the training of a deeper neural network using less training time.

**Table 11:** The prediction results of the final classification models (ensemble model and individual DNN classifier) of the proposed approach using the PSO algorithm and random method for the 5-fold cross-validation runs on the MNIST Dataset. (Here, Avg and Std denote the average and standard deviation of the prediction results, Ensemble and Individual denote the prediction results achieved by the ensemble model and the individual DNN classifier, respectively, and Time1 and Time 2 denote the computational times of the training phases of the ensemble model and the individual DNN classifier, respectively.).

	The proposed approach using PSO				The proposed approach without PSO			
Folds	Ensemble	Individual	Time1	Time2	Ensemble	Individual	Time1	Time2
Fold-1	0.9855	0.9771	987.8683	47.9775	0.9833	0.9650	1016.8787	68.6839
Fold-2	0.9852	0.9771	1209.7731	53.7977	0.9818	0.9794	845.4705	35.4647
Fold-3	0.9832	0.9799	1001.8159	54.6867	0.9821	0.9729	645.3766	24.2177
Fold-4	0.9823	0.9752	1160.9647	75.6790	0.9782	0.9743	655.7204	28.1844
Fold-5	0.9863	0.9792	925.5798	49.0980	0.9859	0.9756	815.5113	33.0172
Avg	0.9845	0.9777	1057.2004	56.2478	0.9823	0.9734	795.7915	37.9136
Std	0.0015	0.0017	108.8520	10.0551	0.0025	0.0047	137.0992	15.8683

Table 12 shows the classification accuracies of the ensemble models with different numbers of DNN classifiers generated by the DNN-PSO and DNN-NONPSO approaches for five runs of 5-fold cross-validation on the MNIST datasets. As shown in the table, higher classification performance is achieved by increasing the DNN classifiers. This demonstrates the advantages of combining evidence across multiple independent classifiers for generalization performance. In addition, to compare the classification accuracies between the networks with two and three hidden layers, the DNN classifiers generated by the solutions (*pbest*) of the PSO algorithm provide significant improvements compared with the random approach, at each fold of the 5-fold cross-validation runs. The proposed approach provides a flexible manner by which the number of classifiers can be determined. In addition, constructing an ensemble model with a certain number of classifiers is directly choosing the optimal classifiers with better scores (low training errors) from all the trained classifiers. In this manner, the generalization capability can be maximized, while the computational time of the prediction phase can be reduced. Therefore, the choice of number of classifiers is a trade-off between performance and complexity. In the next subsection, we investigate the influence of the choice of number of classifiers and evaluate these ensemble models in terms of classification performance and computational time for the prediction phase.

**Table 12:** The prediction results when using ensemble models with different numbers of classifiers.

	Ensemble model using PSO					Ensemble model without PSO				
N of c	Fold-1	Fold-2	Fold-3	Fold-5	Fold-5	Fold-1	Fold-2	Fold-3	Fold-4	Fold-5
2	0.9790	0.9765	0.9766	0.9728	0.9781	0.9273	0.9672	0.9748	0.9686	0.9776
4	0.9818	0.9810	0.9808	0.9798	0.9834	0.9709	0.9777	0.9796	0.9753	0.9825
6	0.9829	0.9829	0.9824	0.9809	0.9853	0.9755	0.9807	0.9812	0.9756	0.9845
8	0.9836	0.9833	0.9823	0.9812	0.9854	0.9768	0.9811	0.9812	0.9758	0.9848
10	0.9843	0.9839	0.9826	0.9818	0.9855	0.9793	0.9820	0.9816	0.9774	0.9842
12	0.9848	0.9844	0.9826	0.9818	0.9860	0.9805	0.9814	0.9822	0.9777	0.9844
14	0.9848	0.9847	0.9827	0.9818	0.9861	0.9814	0.9811	0.9817	0.9782	0.9854
16	0.9851	0.9852	0.9825	0.9819	0.9859	0.9823	0.9822	0.9820	0.9783	0.9855
18	0.9851	0.9849	0.9831	0.9821	0.9863	0.9829	0.9821	0.9826	0.9784	0.9856
Avg	0.9835	0.9830	0.9818	0.9805	0.9846	0.9273	0.9672	0.9748	0.9686	0.9776
Std	0.0020	0.0027	0.0020	0.0030	0.0026	0.9709	0.9777	0.9796	0.9753	0.9825

**Fig 9:**
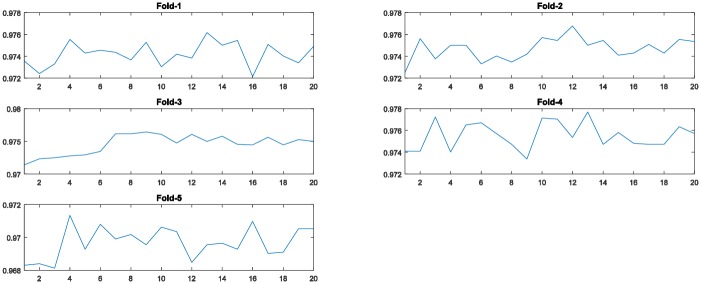
The training accuracy (fitness value) of the PSO algorithm at each generation across all generations for the 5-fold cross-validation runs on the MNIST dataset. (The individual DNN classifier is constructed by three hidden layers. The horizontal axis and the vertical axis represent the number of generations of the PSO algorithm and the training accuracy, respectively.).

**Fig 10:**
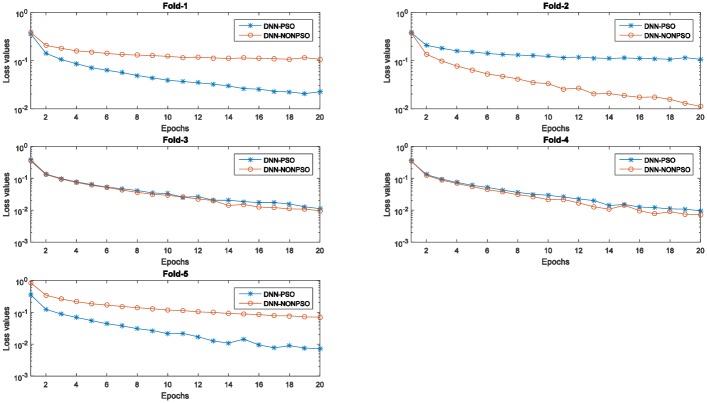
The training error values (loss values) of the network training using three hidden layers with 20 epochs of the individual classifier using the PSO algorithm and random method for five runs of 5-fold cross-validation on the MNIST dataset.

Fig 9 displays the fitness value (training accuracy) curves generated by the PSO algorithm at each iteration for five runs of 5-fold cross-validation on the MNIST dataset. As shown in these graphs, the fitness values gradually increase as the iterations increase, up to a certain number. This indicates that a good solution can be found during the PSO search processes. Fig 10 displays the training error curves generated by the individual DNN classifier using the DNN-PSO and DNN-NONPSO approaches. It can be observed that the DNN-PSO approach almost achieved lower training error curves than DNN-NONPSO during each fold of the 5-fold cross-validation runs.

### 4.5 The influence of the choice of number of classifiers

**Fig 11:**
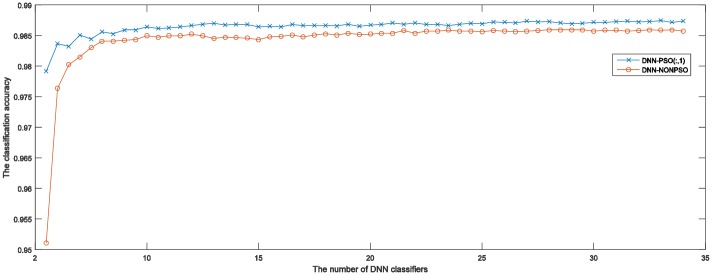
The classification accuracies achieved by various ensemble models with different numbers of classifiers (2 to 59) generated using the DNN-PSO and DNN-NONPSO approaches.

**Fig 12:**
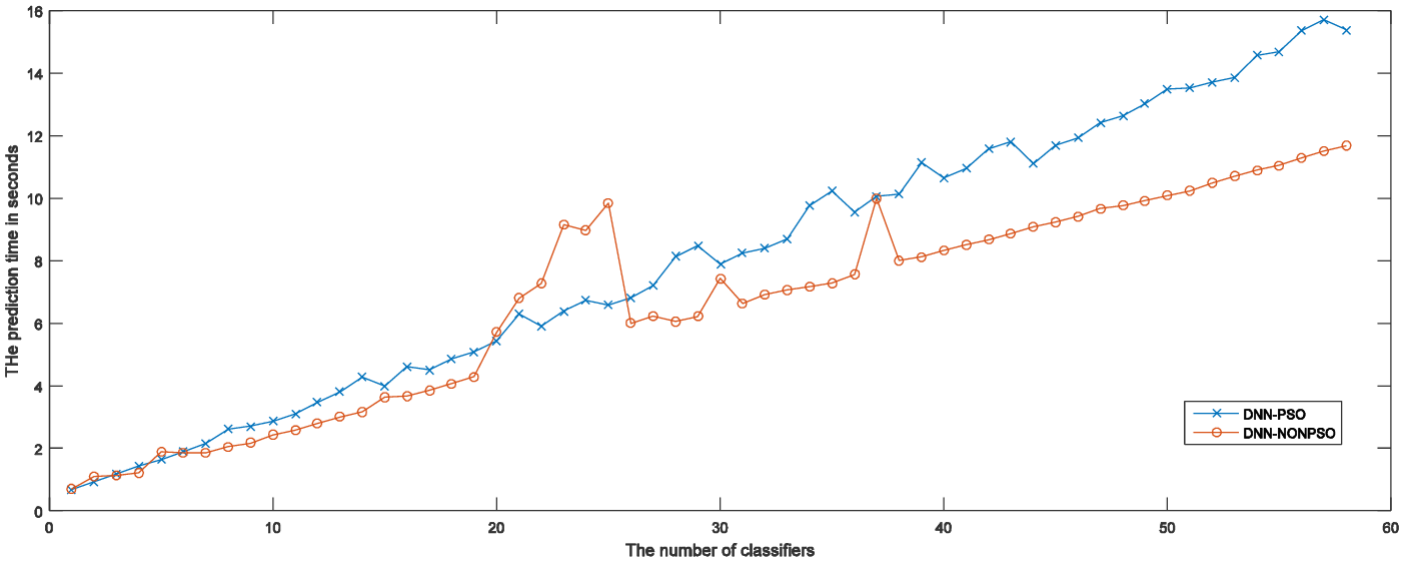
The computational times for the prediction phases of various ensembles with different numbers of classifiers generated using the DNN-PSO and DNN-NONPSO approaches.

**Fig 13:**
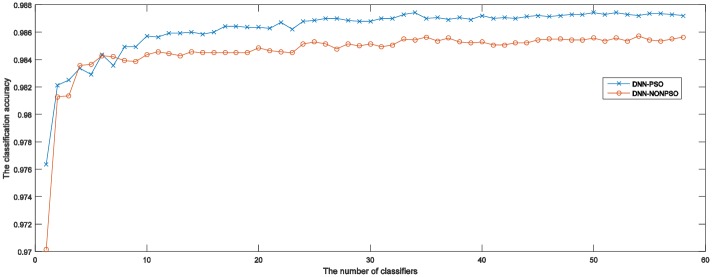
The classification accuracies achieved by various ensemble models based on three hidden layers with different numbers of classifiers (2 to 59) generated using the DNN-PSO and DNN-NONPSO approaches.

**Fig 14:**
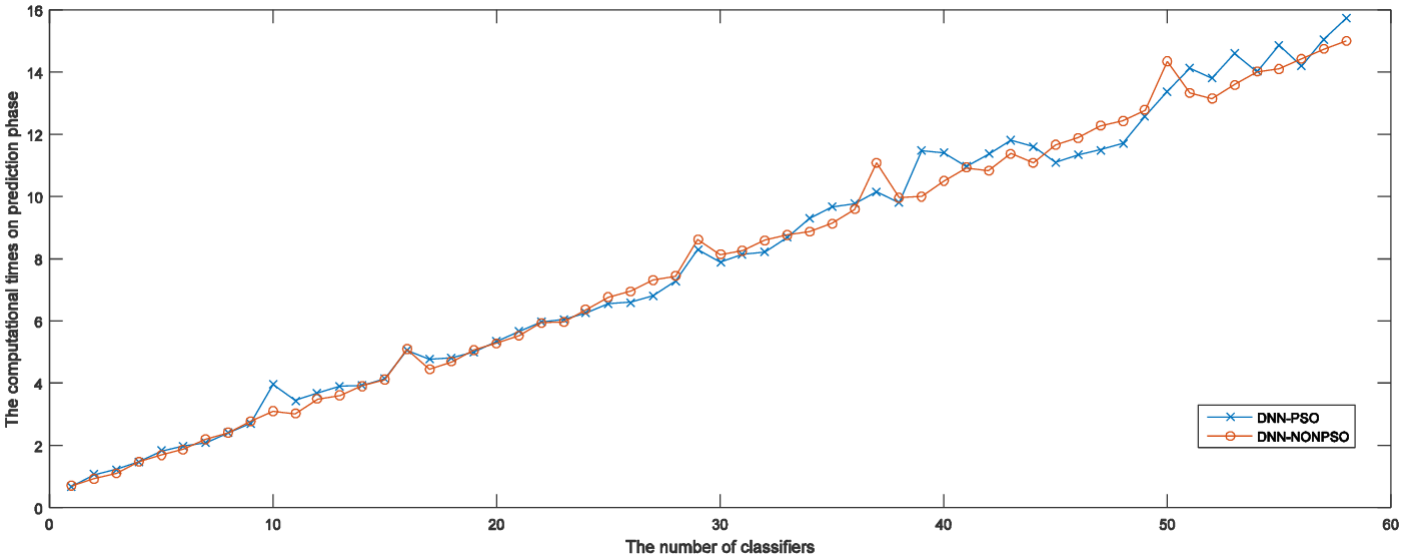
The computational times for the prediction phases of various ensembles based on three hidden layers with different numbers of classifiers generated using the DNN-PSO and DNN-NONPSO approaches.

When constructing an ensemble model, the choice of number of classifiers is an important issue that influences the generalization performance. How to choose a more appropriate number of classifiers is a challenging task because more classifiers may yield better generalization performance but also require more computational resources in network training and prediction. This subsection investigates the influence of the ensemble models that were constructed with different numbers of classifiers using the DNN-PSO and DNN-NONPSO approaches. In this experiment, all parameter configurations are set the same as before expect for the population size (the number of all final classifiers) of the PSO algorithm. In addition, the various ensemble models were generated by directly choosing the classifiers from the candidate classifiers trained by *pbest* of the PSO algorithm and the randomly generated solutions on the MNIST dataset, without training any new neural networks. Fig 11 displays the classification accuracies achieved by the ensemble models with varying numbers of classifiers (from 2 to 59). As shown in the graph, higher classification accuracy can be obtained when more DNN classifiers are employed to construct the ensemble model. In addition, DNN-PSO almost yielded the best classification performance compared with DNN-NONPSO when the ensemble model was constructed by choosing the same number of classifiers. Fig 12 displays the computational times for the training phase of the ensemble models generated by using the DNN-PSO and DNN-NONPOS approaches. From the graph, we can observe that the computational times for the training phase of the ensemble model are gradually increasing as the ensemble model increases its number of members. Figs 13 and 14 respectively display the classification accuracy and computational time of the training phase of ensemble models with three hidden layers. There are similar results to be found in these graphs. Based on the above results, it can be concluded that more DNN classifiers employed to construct an ensemble model may provide better generalization capability, but the resulting long computational times in the training and prediction phases make this inapplicable in real-time applications. Actually, the highest classification performance has been achieved by a certain number of classifiers instead of all classifiers, because the improvement in performance of the ensemble model is smaller when increasing classifiers until a certain number of classifiers is reached. To investigate the influence of the use of different numbers and depths of classifiers when constructing the ensemble model, the same experimental processes as for the parameter configuration were performed.

## 5. Conclusion and future work

In this paper, a new automatic hyperparameter selection approach is proposed to find a more appropriate network structure and hyperparameter configuration for deep neural network training. The main ideal of the proposed approach is utilizing the advantages of global and local exploration capabilities from particle swarm optimization (PSO) and the steepest gradient descent algorithm and combining them into a hybrid search procedure. Because the performance of deep network classifiers extremely depends on their network structure and hyperparameter configurations, we aim to optimize these configurations through an efficient parameter optimization scheme using PSO in combination with the steepest gradient descent algorithm. After the procedure of the parameter optimization scheme, the final solutions (pbest) and (gbest) of the PSO algorithm having important network configuration information are used to initialize and train a final ensemble model and individual DNN classifier, respectively. In addition, the proposed approach also provides a flexible method that allows users to choose a certain threshold as the number of classifiers to construct a light ensemble model. In this manner, the trade-off between the generalization capability and the model complexity can be addressed, and this is discussed in the previous subsections in which several experiments have been performed with the ensemble models with different numbers of classifiers. We have constructed experimental studies to solve classification and regression problems by evaluating the performance of the proposed approach on the handwritten characters and biological activity prediction datasets, respectively. The experimental results demonstrated that the proposed approach can find a more appropriate network structure with a better hyperparameter configuration, which are then employed to initialize and train DNN classifiers, which achieve excellent performance in both the training phase and final models (after training). Therefore, the proposed approach can be regarded as an automatic hyperparameter optimization tool for deep learning architectures without requiring any prior knowledge.

In our future work, we would like to extend our approach to optimize more deep learning architectures, such as convolutional neural networks (CNNs) and recurrent neural networks (RNNs), since the flexibility of the proposed approach in initializing and training a neural network means that the above implementation would require only a small modification. In addition, we would like to use other advanced evolutionary algorithms or develop a new algorithm to replace PSO in the proposed approach for network configuration optimization since it provides a flexible optimization scheme in which the algorithm is performed in a wrapped manner, and any population-based optimization algorithm can be employed.
